# Physical Exercise Prevented Stress‐Induced Anxiety via Improving Brain RNA Methylation

**DOI:** 10.1002/advs.202105731

**Published:** 2022-06-01

**Authors:** Lan Yan, Ji‐an Wei, Fengzhen Yang, Mei Wang, Siqi Wang, Tong Cheng, Xuanjun Liu, Yanbin Jia, Kwok‐Fai So, Li Zhang

**Affiliations:** ^1^ Key Laboratory of Central CNS Regeneration (Ministry of Education), Guangdong‐Hong Kong‐Macau Institute of CNS Regeneration Jinan University Guangzhou 510632 P. R. China; ^2^ College of Life Science and Technology Jinan University Guangzhou 510632 P. R. China; ^3^ Department of Psychiatry, The First Affiliated Hospital Jinan University Guangzhou 510632 P. R. China; ^4^ Institute of Clinical Research for Mental Health, The First Affiliated Hospital Jinan University Guangzhou 510632 P. R. China; ^5^ State Key Laboratory of Brain and Cognitive Science, Li Ka Shing Faculty of Medicine The University of Hong Kong Hong Kong SAR P. R. China; ^6^ Center for Brain Science and Brain‐Inspired Intelligence Guangdong‐Hong Kong‐Macao Greater Bay Area Guangzhou 510515 P. R. China; ^7^ Co‐Innovation Center of Neuroregeneration Nantong University Jiangsu 226019 P. R. China; ^8^ Neuroscience and Neurorehabilitation Institute University of Health and Rehabilitation Sciences Qingdao 266000 P. R. China

**Keywords:** anxiety disorder, exercise training, liver metabolism, medial prefrontal cortex, RNA methylation

## Abstract

Physical exercise is effective in alleviating mental disorders by improving synaptic transmission; however, the link between body endurance training and neural adaptation has not yet been completely resolved. In this study, the authors investigated the role of RNA N^6^‐methyladenosine (m6A), an emerging epigenetic mechanism, in improved resilience against chronic restraint stress. A combination of molecular, behavioral, and in vivo recording data demonstrates exercise‐mediated restoration of m6A in the mouse medial prefrontal cortex, whose activity is potentiated to exert anxiolytic effects. Furthermore, it is revealed that hepatic biosynthesis of one methyl donor is necessary for exercise to improve brain RNA m6A to counteract environmental stress. This novel liver‐brain axis provides an explanation for brain network changes upon exercise training and provides new insights into the diagnosis and treatment of anxiety disorders.

## Graphical abstract

N^6^‐methyladenosine (m6A) is one common posttranscriptional modification of mRNA molecules and is related with neural functions. Aerobic exercise can elevate the hepatic synthesis of methyl donor molecules, which potentiate the m6A of synaptic transcripts in medial prefrontal cortex to counteract stress‐induced anxiety. The current findings uncover a liver‐brain axis under exercise paradigms to mediate central epigenetics and mental functions.

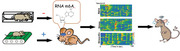

## 1 Introduction

Repeated environmental stress induces anxiety disorders in both rodents^[^ ^1^, ^2^ ^]^ and humans.^[^ ^3^ ^]^ Current studies suggest the involvement of both cortical and subcortical nuclei in the anxiety circuit.^[^ ^4^, ^5^ ^]^ Among these, the medial prefrontal cortex (mPFC) works as a higher center for mental and cognitive regulation and presents with dysfunctions upon chronic stress.^[^ ^6^, ^7^, ^8^, ^9^ ^]^ Although major progress has been made regarding the neural circuits governing anxiety disorders,^[^ ^10^ ^]^ the molecular mechanisms that explain how environmental stress can be translated into disrupted neural transmission remain incomplete. As suggested by a recent study, epigenetic modulations, including N^6^‐methyladenosine (m6A) of RNA, are altered upon acute stress.^[^ ^11^ ^]^ However, no systemic study has been performed to investigate the m6A profile of the mPFC under chronic stress.

Physical exercise training effectively ameliorates anxiety disorders.^[^ ^12^, ^13^ ^]^ When exploring the neural mechanism of exercise, classical views argue for the potentiation of adult hippocampal neurogenesis downstream of neurotrophic factors.^[^ ^14^, ^15^, ^16^ ^]^ In the cortical region, we recently showed that repeated treadmill exercise facilitates spinogenesis and stabilization of newly formed synapses.^[^ ^17^, ^18^, ^19^ ^]^ However, there is a major gap between exercise training and behavioral improvements due to the incomplete picture of how exercise is translated into synaptic modulations across multiple brain regions. To provide molecular explanations, it is worth noting that epigenetic regulation, such as histone modification,^[^ ^20^, ^21^ ^]^ microRNA,^[^ ^22^ ^]^ and DNA methylation^[^ ^23^, ^24^ ^]^ all participate in mental and cognitive rehabilitation following exercise. Nevertheless, as a post‐transcriptional regulatory pathway that prominently affects synaptic function and memory,^[^ ^25^, ^26^ ^]^ the potential role of RNA m6A in exercise‐mediated anxiolytic effects has not yet been investigated.

In the current study, we adopted a 14‐day chronic restraint stress (CRS) paradigm to generate anxiety‐like behaviors in adult mice,^[^ ^18^ ^]^ in which RNA m6A homeostasis was disrupted in the mPFC and was restored by exercise training to exert anxiolytic effects. Further analysis showed that exercise potentiated m6A on excitatory synapse‐related genes and activated mPFC neurons along with its projection toward the basolateral amygdala (BLA), thus conferring stress resilience. As a peripheral mechanism, we identified the methyl donor S‐adenosyl methionine (SAM), the biosynthesis of which was potentiated in liver tissues after exercise, contributing to the restoration of brain RNA methylation, neural activity, and anxiolytic effects. Taken together, this liver‐brain axis provides an epigenetic mechanism for brain adaptation following exercise to counteract environmental stress.

## 2 Results

### 2.1 Physical Exercise Enhances Stress Resilience via Potentiating Brain RNA Methylation

**Figure 1:**
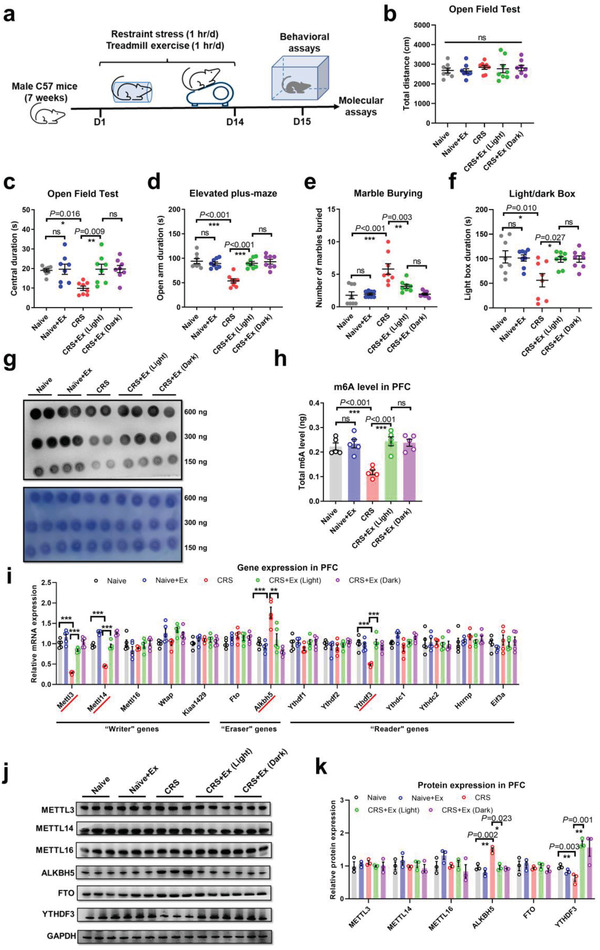
Treadmill exercise relieves anxiety‐like behaviors and restores brain RNA m6A levels. a) Experimental schedules. Chronic restraint stress (CRS) and treadmill exercise were performed on male wild‐type mice for 14 days, followed by behavioral assays. b) No significant change of total distance moved in the open field when mice were subjected to exercise during the light (ZT 0200–0300) or dark (ZT 1400–1500) cycle. One‐way ANOVA, *F*(4,35) = 0.3185, *P* = 0.864. c) Central duration in the open field was lower following CRS and re‐elevated following exercise during the light or dark phase. One‐way ANOVA, *F*(4,35) = 4.966, *P* = 0.003. d) CRS mice spent less time in the open arm on the elevated plus‐maze, and exercise recovered normal behavior. One‐way ANOVA, *F*(4,35) = 15.56, *P* < 0.001. e) CRS animals displayed more repetitive behaviors in the marble burying paradigm, which was normalized according to treadmill exercise. One‐way ANOVA, *F*(4,35) = 13.23, *P* < 0.001. f) CRS enhanced avoidance of the light box, and exercise training in either the light or dark phase recovered normal preference levels. One‐way ANOVA, *F*(4,35) = 4.400, *P* = 0.006. *N* = 8 mice in each group in b–f). g) Dot‐blotting assays for the content of RNA‐m6A. Twofold serial dilution of RNA samples were loaded. Methylene blue staining (lower panels) was used as a loading control. h) Total m6A amount (in ng) in prefrontal cortex (PFC) tissue extracts was depressed following CRS and elevated following physical exercise. One‐way ANOVA, *F*(4,20) = 12.40, *P* < 0.001. i) Quantification of relative gene expression of RNA methylation regulators, including methyltransferase (“Writer”), demethylase (“Eraser”), and RNA binding proteins (“Reader”). Multiple *t*‐test showed significant changes in *Mettl3* (*P* < 0.001), *Mettl14* (*P* < 0.001), *Alkbh5* (*P* < 0.001), and *Ythdf3* (*P* < 0.001) genes, and no significant difference in all other genes (*P* > 0.05). *N* = 5 mice in each group in h,i). j) Representative blotting bands for major RNA methylation regulators from PFC extracts. k) Quantification analysis showed CRS‐induced expression of ALKBH5, which was depressed by exercise training. YTHDF3 was potentiated by exercise training. Multiple *t*‐tests showed significant changes in both ALKBH5 (*P* < 0.001) and YTHDF3 (*P* < 0.001) proteins, but not for other proteins (*P* > 0.05). *N* = 3 mice in each group. ns, no significant difference; *P < 0.05, **P < 0.01, ***P < 0.001 using Tukey's post‐hoc comparisons. All data are presented as mean±SEM.

We first described brain RNA m6A homeostasis in the mouse PFC under environmental stress conditions. Using a single bout of physical restraint, adult male mice exhibited anxiety‐like behaviors (Extended Data Figure S1a–d, Supporting Information), in conjunction with lower m6A levels in the PFC (Extended Data Figure S1e, Supporting Information). These results were in line with the decreased expression level of one RNA methylase, *Mettl3* (Extended Data Figure S1f, Supporting Information). These data agree with previous findings^[^ ^18^ ^]^ and show brain RNA demethylation upon environmental stress. To study the role of m6A in exercise‐mediated anxiolysis, we subsequently employed a 14‐day CRS scheme with 1 h of daily treadmill exercise (**Figure** **1**a). Behavioral phenotyping suggested that exercise, either in the light or dark phase, did not alter the overall locomotor activity in the open field (Figure 1b), but conferred resilience against CRS‐induced anxiety‐like behaviors (Figure 1c–f). Specifically, exercise in naïve animals did not alter behavioral phenotyping, suggesting that treadmill running mainly exerted anxiolytic effects. In parallel with behavioral modulations, exercise also helped to restore m6A contents in the PFC in CRS‐treated mice (Figure 1g–h), while leaving m6A content in the hippocampus or BLA unchanged (Extended Data Figure S2a, Supporting Information). The restoration of RNA methylation by exercise might be explained by the elevated expression of RNA methylase (*Mettl3*, *Mettl14*, *Mettl16*)^[^ ^27^ ^]^ and downregulation of RNA demethylase (*Alkbh5*),^[^ ^28^ ^]^ in addition to elevated expression of the RNA‐binding factor *Ythdf3* ^[^ ^29^ ^]^ (Figure 1i). Further protein quantification assays confirmed that exercise training repressed ALKBH5 in the PFC (Figure 1j,k), contributing to elevated m6A levels.

**Figure 2:**
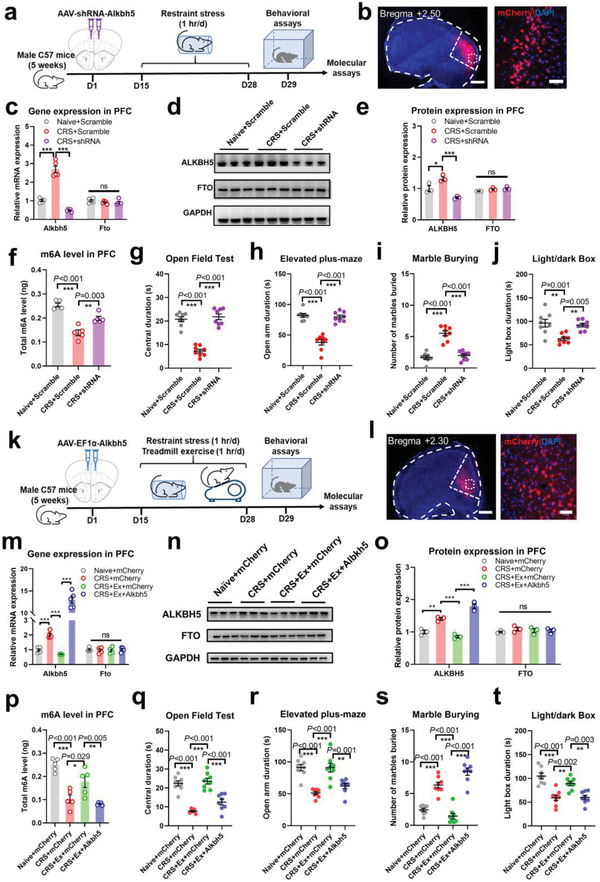
Brain RNA methylation plays a role in exercise‐conferred stress resilience. a) Timeline of experiments demonstrating the necessity of m6A in stress resilience. Mice received bilateral injection of adeno‐associated virus (AAV) carrying short hairpin RNA (shRNA) of RNA demethylase, *Alkbh5*, into the prelimbic (PrL) region, followed by 14‐day CRS and behavioral phenotyping. b) Immunofluorescent images of viral transfection. Scale bar, 300 µm in left panel or 100 µm in right panel. c) Relative gene expression of *Alkbh5* and *Fto* in PFC extracts. Multiple *t*‐tests showed significant change in *Alkbh5* (*P* < 0.001) but not *Fto* gene expression (*P* = 0.202). *N* = 5 mice in each group d) Protein blotting bands for ALKBH5 and FTO. e) Quantification of relative protein expression. Multiple *t*‐tests showed CRS‐ and shRNA‐mediated changes of ALKBH5 (*P* < 0.05) but not in FTO proteins. *N* = 3 mice in each group. f) Total m6A contents in the PFC was elevated by suppressing *Alkbh5* gene expression. One‐way ANOVA, *F*(2,12) = 34.94, *P* < 0.001. *N* = 5 mice in each group. g) Suppressing *Alkbh5* expression increased the duration spent in the center of the open field arena. *F*(2,21) = 62.69, *P* < 0.001. h) *Alkbh5* gene knockdown in mPFC increased time spent in the open arm on the elevated plus‐maze. *F*(2,21) = 48.62, *P* < 0.001. i) Suppressing *Alkbh5* gene expression inhibited repetitive behaviors. *F*(2,21) = 46.06, *P* < 0.001. j) Decreased RNA demethylase expression also relieved light box avoidance in CRS mice. *F*(2,21) = 10.15, *P* < 0.001. *N* = 8 mice in each group in g–j). k) Schematic illustrations of experiments for the role of m6A in exercise‐mediated anxiolytic effects. Fourteen days after bilateral injection of AAV expressing the *Alkbh5* gene, mice underwent CRS and treadmill exercise paradigms. l) Immunofluorescent images showing viral transfection site. Scale bar, 300 µm in left panel or 100 µm in right panel. m) Relative expression of *Alkbh5* and *Fto* gene transcripts. Multiple *t*‐tests showed the mediation of *Alkbh5* (*P* < 0.001) but no compensatory effect of *Fto* (*P* = 0.728) on *Alkbh5* overexpression. *N* = 5 mice in each group. n) Western blotting bands showing ALKBH5 and FTO protein expression. o) Quantification of protein expression level. Multiple *t*‐tests showed remarkable changes in ALKBH5 upon AAV transfection (*P* < 0.001) but no change in FTO. *N* = 3 mice in each group. p) Total brain m6A level was decreased by overexpressing *Alkbh5*. One‐way ANOVA, *F*(3,16) = 20.59, *P* < 0.001. *N* = 5 mice in each group. q) Suppressing m6A decreased time spent in the central field of the open arena in exercised mice. *F*(3, 28) = 35.92, *P* < 0.001. r) Time spent in the open arm of the elevated plus‐maze decreased after overexpression of *Alkbh5*. *F*(3,28) = 17.69, *P* < 0.001. s) *Alkbh5* overexpression induced more marble burying behavior. *F*(3,28) = 53.54, *P* < 0.001. t) Exercise‐relieved light avoidance was abolished by *Alkbh5* transfection. *F*(3,28) = 17.98, *P* < 0.001. *N* = 8 mice in each group in q–t). ns, no significant difference; *P < 0.05, **P < 0.01, ***P < 0.001 using Tukey's post‐hoc comparisons. All data are presented as mean±SEM.

Next, we aimed to illustrate the role of RNA m6A in maintaining stress resilience. Bilateral transfection of adeno‐associated virus (AAV)‐mediated overexpression of *Alkbh5* into the prelimbic region (PrL), remarkably suppressing m6A levels (Extended Data Figure S2b–e, Supporting Information). Although these mice presented with normal growth and motor activities (Extended Data Figure S2f,g, Supporting Information), they displayed remarkable anxiety‐like features (Extended Data Figure S2h,i, Supporting Information). In contrast, short hairpin RNA (shRNA)‐mediated silencing of *Alkbh5* effectively elevated PFC m6A levels in CRS mice without affecting the expression of another RNA demethylase *Fto* (**Figure** **2**a–f), resulting in the relief of anxiety‐like behaviors (Figure 2g–j). Such data recapitulated the beneficial effects of exercise training, suggesting that RNA methylation in the PFC is necessary in conferring stress resilience to prevent anxiety‐like disorders.

Finally, the necessity of brain RNA m6A to exercise‐mediated anxiolytic effects was tested. Under AAV‐mediated overexpression of *Alkbh5* (Figure 2k–o), mice presented with lower levels of total m6A in the PFC, even with the exercise intervention (Figure 2p) and displayed anxiety‐like behaviors (Figure 2q–t). Therefore, suppression of brain m6A effectively abolished the beneficial effects of exercise training in preventing anxiety‐like behaviors. To further delineate the role of RNA methylation, rather than *Alkbh5* alone in exercise‐conferred stress resilience, the expression of m6A‐associated RNA binding factor *Ythdf3* was further silenced by shRNA under a Tet‐On system composed of a TRE3g promoter that requires the rtTA transcription factor that can be activated by doxycycline (DOX). The induced repression of *Ythdf3* (Extended Data Figure S2j–l, Supporting Information) remarkably abolished exercise‐related anxiolytic effects without affecting local m6A levels (Extended Data Figure S2m–o, Supporting Information). These data collectively suggest that exercise‐conferred stress resilience is dependent on RNA methylation in the mPFC.

### 2.2 Exercise Mediates m6A of Excitatory Synapse‐Related Transcripts to Potentiate Cortical Activity

**Figure 3:**
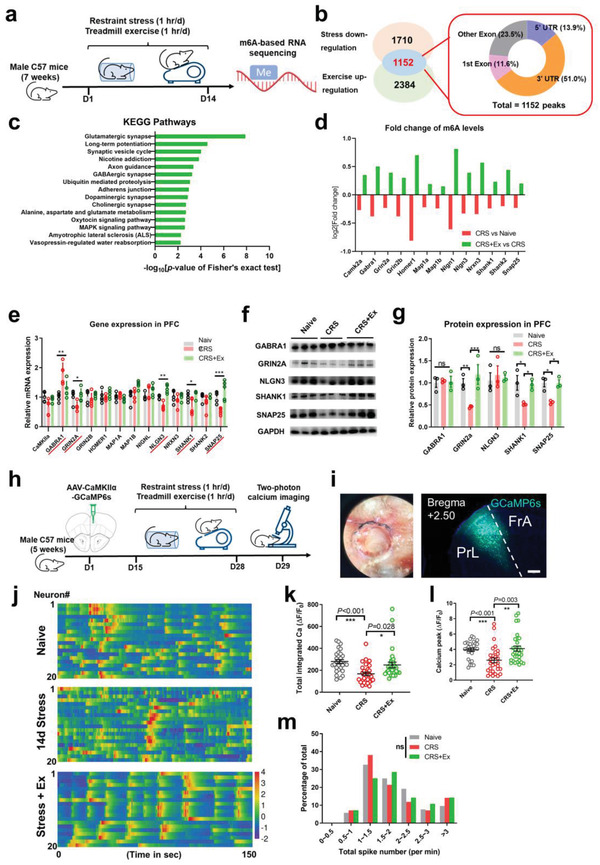
Exercise modulates brain RNA m6A to enhance cortical activities. a) Experimental protocols for m6A‐based immunoprecipitation (IP) RNA sequencing. b) Left: Venn diagram showed a total of 1152 sites that were coregulated by CRS and exercise. Right: Distribution of target sites with respect to transcript domain. More than half of the m6A sites were located on 3′‐untranslated regions (3′UTR). c) The top 15 pathways of the m6A peaks in the Kyoto Encyclopedia of Genes and Genomes (KEGG) database ranked by *P* value. d) A list of synapse‐associated genes containing m6A peaks, as presented by the averaged fold change in m6A levels (normalized to naïve group). C, control (naïve) group; S, chronic restraint stress (CRS) group; RS, CRS + treadmill running group. *N* = 3 biological triplicates for each group of m6A‐IP sequencing, with three animals pooled in biological sample. e) Relative gene expression level of synaptic genes in PFC tissues. Multiple *t*‐tests showed significant changes in GABA_A_ receptor *α*1 (GABRA1), glutamate ionotropic receptor NMDA type subunit 2A (GRIN2a), Neuroligin 3 (NLGN3), SH3, and multiple ankyrin repeat domains 1 (SHANK1), and synaptosome associated protein 25 (SNAP25). *N* = 5 mice in each group. f) Protein blotting bands of five synaptic components. g) Relative protein expression of f). Multiple *t*‐tests showed exercise‐mediation of GRIN2a, SHANK1, and SNAP25. *N* = 3 mice in each group. h) Schematic illustrations of two‐photon recording. Mice received unilateral injection of genetically encoded fluorescent calcium indicator GCaMP6s into the PrL region, followed by 14 d CRS and exercise. Two‐photon in vivo calcium imaging was performed 24 h after the conclusion of the exercise paradigm. i) Left, bright field view of transcranial imaging window on top of the PrL. Right, fluorescent imaging of coronal sections showing GCaMP6s expression. Scale bar, 250 µm. j) Heatmap of relative calcium fluorescence intensity, with 20 representative neurons in each group. k) Total integrated calcium levels were depressed following CRS and recovered following exercise training. Kruskal–Wallis statistic = 20.77, *P* < 0.001. l) The peak value of each calcium transient showed similar patterns of change. Kruskal–Wallis statistic = 16.51, *P* < 0.001. m) Distribution of calcium transient frequency (spike per min) showed unchanged calcium spiking numbers. Kruskal–Wallis statistic = 4.314, *P* = 0.116. *n* = 30 neurons from four mice in each group in k–m). ns, no significant difference, *P < 0.05, **P < 0.01, ***P < 0.001 using Tukey's post‐hoc comparisons in e,g) and using Dunn's multiple comparison test in k–m). All data are presented as mean±SEM.

Having acknowledged the necessary role of exercise‐mediated brain RNA m6A in stress resilience, we next investigated m6A‐targeted transcripts. Using an m6A immunoprecipitation (IP)‐based RNA sequencing approach on triplicated brain samples,^[^ ^30^ ^]^ we identified a total of 1152 sites whose m6A levels were downregulated by CRS and upregulated under exercise intervention (**Figure** **3**a,b; and Extended Data Table S1, Supporting Information), making them potential candidates for molecular substrates of exercise training. Bioinformatics analysis using the Kyoto Encyclopedia of Genes and Genomes database revealed that these transcripts were enriched in synapse‐related pathways (Figure 3c), which involved structural and functional genes related to synaptic plasticity (Figure 3d; Extended Data Table S1, Supporting Information). Further molecular studies revealed the altered expression of excitatory synaptic proteins, including glutamate ionotropic receptor NMDA type subunit 2A (GRIN2a), SH3 and multiple ankyrin repeat domains 1 (SHANK1), and synaptosome associated protein 25 (SNAP25; Figure 3e–g). In agreement with previous findings showing highly dynamic m6A levels of synapse‐related transcripts,^[^ ^31^ ^]^ our sequencing results support systemic modulation by exercise training in improving stress resilience.

To provide functional evidence of the epigenomic and molecular data, we characterized the neuronal activity in PrL using immediate early genes (IEGs) and found repotentiation of neurons following exercise training (Extended Data Figure S3a,b, Supporting Information). Furthermore, we employed an in vivo imaging approach to monitor the calcium activity of PrL neurons via stereotaxic transfection of genetically coded fluorescent calcium indicator GCaMP6s, followed by two‐photon imaging in awake and head‐fixed mice (Figure 3h,i). The calcium activity of pyramidal neurons in the PrL was remarkably depressed following 14‐day CRS and re‐elevated following the exercise intervention (Figure 3j,k). Both CRS and exercise mainly affected the peak value of a single calcium spike rather than its frequency (Figure 3l,m), indicating postsynaptic potentiation. To test whether exercise‐driven anxiolytic effects were dependent on PrL neuronal activation, we employed chemogenetic approaches to manipulate PrL activity. Neuronal activation through the designer receptor exclusively activated by designer drugs (DREADD) receptor, hM3Dq, did not affect brain m6A levels (Extended Data Figure S3c–f, Supporting Information) but effectively relieved anxiety‐related behaviors in CRS mice, largely recapitulating exercise effects (Extended Data Figure S3g–h, Supporting Information). However, the inhibition of cortical neurons using the hM4Di receptor abolished exercise‐induced stress resilience (Extended Data Figure S3i–n, Supporting Information). These data collectively suggest that m6A‐mediated cortical excitation underlies exercise‐related anxiolytic effects.

### 2.3 Exercise Drives RNA m6A to Activate a Cortico‐Amygdala Circuit for Conferring Stress Resilience

**Figure 4:**
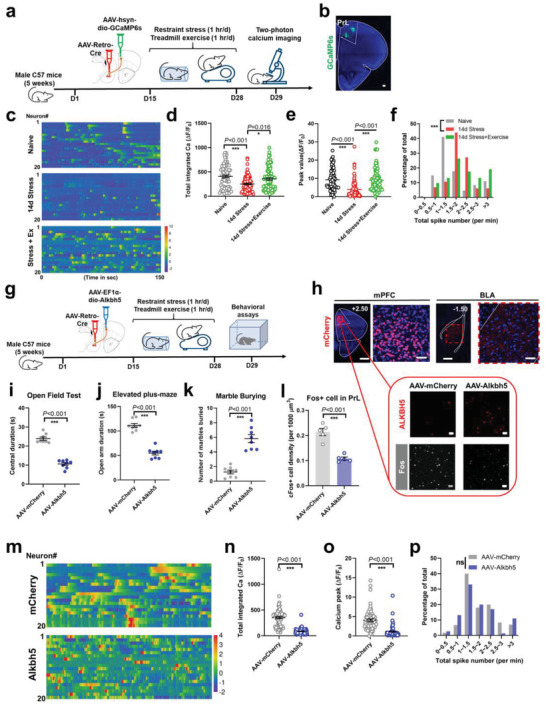
A cortico‐amygdala pathway underlies m6A‐mediated stress resilience. a) Experimental protocols for BLA‐projecting cortical neuron recording. One AAV‐Retro vector expressing Cre recombinase was injected into the anterior BLA, followed by the transfection of DIO‐driven GCaMP6s into the PrL region. Two‐photon in vivo calcium imaging was performed after 14 days of CRS and treadmill exercise. b) Coronal sections showing the labeling of BLA‐projecting neurons in the PrL. Scale bar, 100 µm. c) Heatmap of relative calcium fluorescence intensity as displayed by z‐score. A total of 20 representative neurons were shown in each group. d) Total integrated calcium activities of BLA‐projecting pyramidal neurons in the PrL was suppressed by CRS and recovered by exercise training. Kruskal–Wallis statistic = 17.55, *P* < 0.001. e) CRS depressed peak values of calcium transients, which were recovered by exercise training. Kruskal–Wallis statistic = 55.93, *P* < 0.001. f) Frequency of calcium transients was depressed by CRS and unchanged by exercise training. Kruskal–Wallis statistic = 82.45, *P* < 0.001. *n* = 80 neurons from five mice in each group in d–f). g) Experimental timelines for manipulating m6A in cortico‐amygdala circuit. Cre‐dependent *Alkbh5*‐expressing AAV vector was injected into the PrL with AAV‐Retro‐Cre‐mCherry into the BLA. h) Upper panels, transfected neurons in the PrL (left), and their projection terminus in the BLA (right). Lower panels, immunofluorescent staining for *Alkbh5* (upper) and Fos (lower) in the PrL. Scale bar, 500 µm in panoramic views and 80 µm in enlarged images. i–k) Enhancing *Alkbh5* expression in BLA‐projecting PrL neurons decreased central duration in the open field (two‐sample *t*‐test, *t*(14) = 11.67, *P* < 0.001; i), decreased time spent in the open arm in the elevated plus‐maze (two‐sample *t*‐test, *t*(14) = 10.17, *P* < 0.001; j), and induced more repetitive behaviors (two‐sample *t*‐test, *t*(14) = 7.563, *P* < 0.001; k). *N* = 8 mice in each group. l) Quantification of Fos immunoreactivity showed suppressed cortical activity by potentiating demethylation. Two‐sample *t*‐test, *t*(20) = 6.147, *P* < 0.001. *n* = 11 slices from four animals in each group. m) Heatmap of relative calcium fluorescence intensity, with a total of 20 representative neurons in each group. Data were normalized as z‐scores. n) RNA demethylation suppressed total integrated calcium activities in BLA‐projecting neurons. Kolmogorov–Smirnov *D* = 0.8250, *P* < 0.001. o) Overexpressing *Alkbh5* also depressed peak values of calcium transients. Kolmogorov–Smirnov *D* = 0.7162, *P* < 0.001. p) Unchanged distribution of calcium transient frequency. Kolmogorov–Smirnov *D* = 0.1237, *P* = 0.684. *n* = 80 neurons from five mice in each group in n–p). ns, no significant difference; *P < 0.05, **P < 0.01, ***P < 0.001 using Dunn's multiple comparison test in d–f), two‐sample *t*‐test in i–l), and Kolmogorov–Smirnov test in n–p). All data are presented as mean±SEM.

Recent human studies have shown disrupted functional connectivity of PFC‐related brain networks underlying anxiety disorders.^[^ ^32^, ^33^ ^]^ The PrL‐BLA projection was examined as it is involved in environmental stress‐coping regulation.^[^ ^2^ ^]^ Using a dual‐virus labeling system in which an AAV‐Retro‐Cre vector was transfected into the anterior BLA, followed by a second AAV‐expressing GCaMP6s flanked by a double inverted orientation (DIO) sequence into the PrL region, BLA‐projecting PrL neurons were labeled and recorded in vivo (**Figure** **4**a,b). Similar to whole‐PrL imaging (Figure 3i), BLA‐projecting neurons were inhibited under CRS, and displayed potentiated calcium activity after treadmill exercise (Figure 4d), which was attributed to elevated peak values (Figure 4e). However, exercise training did not change the frequency of calcium transients (Figure 4f). Exercise intervention thus activated the PrL‐BLA circuit, contributing to improved stress resilience.

Whether this circuit is mediated by RNA methylation, which has been demonstrated to be affected by exercise training, was unclear. To address this topic, we modified the RNA m6A manipulation strategy by using an AAV vector carrying *Alkbh5* under the governance of the Ca^2+^/calmodulin‐dependent protein kinase II alpha (CaMKII*α*) promoter, thus achieving excitatory neuron‐specific m6A suppression in PrL (Extended Data Figure S4a–c, Supporting Information). RNA demethylation in these neurons was sufficient to suppress neuronal activity and induce anxiety‐like behaviors (Extended Data Figure S4d–g, Supporting Information). Subsequently, circuit‐specific suppression of neuronal m6A was prepared by transfecting PrL with an AAV expressing *Alkbh5* in a Cre‐dependent manner, following AAV‐Retro‐Cre in BLA (Figure 4g,h). Behavioral phenotyping showed that disruption of m6A specifically in the PrL‐BLA circuit led to anxiety‐related phenotypes (Figure 4i–k), suggesting the dependence of exercise intervention on m6A within this PrL‐BLA circuit. Based on the decreased neuronal activity of PrL after m6A depression (Figure 4h,l), we employed two‐photon imaging of the BLA‐projecting cortical neurons. RNA demethylation remarkably disrupted normal calcium activity, as suggested by weaker calcium peaks and lower frequencies (Figure 4m–p). These observations replicated those patterns under CRS treatment (Figure 4c), suggesting the importance of RNA methylation in this cortico‐amygdala circuit to affect anxiety‐like behaviors.

### 2.4 Hepatic Biogenesis of Methyl Donors Participates in Exercise‐Mediated Anxiolytic Effects

**Figure 5:**
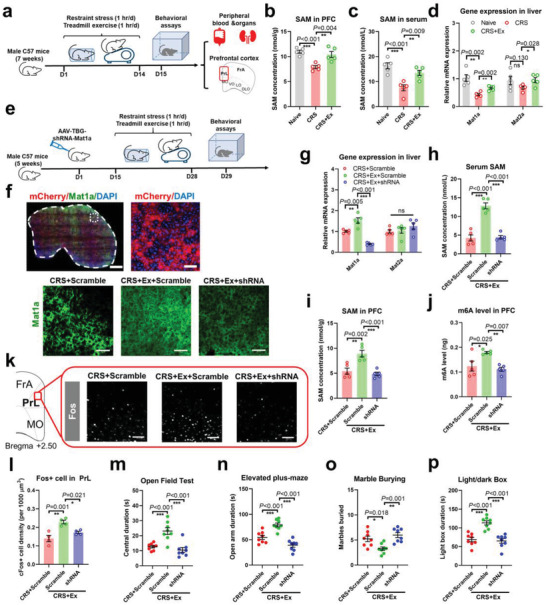
Hepatic biosynthesis of methyl donors potentiated by exercise confers stress resilience. a) Experimental schedules for central and peripheral quantification of S‐adenosyl methionine (SAM). b) SAM concentration in the PFC was suppressed by CRS and potentiated by treadmill exercise. One‐way ANOVA, *F*(2,12) = 14.92, *P* < 0.001. c) Circulating SAM levels followed similar patterns as those in the brain. One‐way ANOVA, *F*(2,12) = 14.66, *P* < 0.001. d) Relative expression of *Mat1a* and *Mat2a* genes in liver tissues. Multiple *t*‐test showed that CRS decreased *Mat1a* (*P* = 0.002) but not *Mat2a* (*P* = 0.130) gene. *N* = 5 mice each group in b–d). e) Schematic illustration of hepatic silencing of SAM biosynthesis. Liver‐targeted AAV vector containing shRNA of *Mat1a* was infused via intravenous injection, followed by CRS and exercise training. f) Fluorescent imaging of liver slices showed the hepatic viral transfection (upper panels) and suppression of *Mat1a* expression (lower panels). Scale bar, 500 µm in panoramic views, and 50 µm in enlarged images. g) Multiple *t*‐tests showed downregulation of *Mat1a* (*P* < 0.001) but not *Mat2a* (*P* = 0.335) in liver tissues after shRNA transfection. h) Hepatic *Mat1a* knockdown repressed the circulating SAM level that was elevated by exercise training in CRS mice. One‐way ANOVA, *F*(2,12) = 48.44, *P* < 0.001. i) Central concentration of SAM was also depressed by hepatic *Mat1a* gene knockdown. *F*(2,12) = 15.35, *P* < 0.001. j) Brain m6A concentration also decreased with liver *Mat1a* deficiency. *F*(2,12) = 8.098, *P* = 0.006. *N* = 5 mice in each group in g–j). k) Immunofluorescent staining of Fos+ cells in PrL. Scale bar, 150 µm. l) *Mat1a* knockdown in the liver remarkably suppressed PrL neuronal activity. *F*(2,9) = 14.24, *P* = 0.002. *N* = 4 mice in each group (averaged from three slices per animal). m) Liver‐specific *Mat1a* knockdown antagonized the effect of exercise in CRS mice and induced anxiety‐like behaviors as displayed by lower time spent in the central arena. *F*(2,21) = 16.30, *P* < 0.001. n) Less time spent in the open arm of the elevated plus‐maze with *Mat1a* deficiency in liver tissues. *F*(2,21) = 28.32, *P* < 0.001. o) *Mat1a* gene knockdown induced more repetitive behaviors. *F*(2,21) = 9.206, *P* = 0.001. p) Hepatic *Mat1a* knockdown abolished exercise‐enhanced light box duration. *F*(2,21) = 21.06, *P* < 0.001. *N* = 8 mice in each group in m–p). ns, no significant difference; *P < 0.05, **P < 0.01, ***P < 0.001 using Tukey's multiple comparison test. All data are presented as mean±SEM.

Demonstrating an epigenetic circuit behavior mechanism underlying exercise‐mediated anxiolytic effects raises an intriguing question about how exercise affected central epigenetic regulation. SAM is a critical methyl donor for mRNA inside the body,^[^ ^34^ ^]^ and endogenous SAM is mainly synthesized by the enzyme methionine adenosyl transferase (MAT).^[^ ^35^ ^]^ *Mat1a* is primarily located in hepatic tissues,^[^ ^36^ ^]^ the metabolic homeostasis of which is dramatically affected by exercise training.^[^ ^37^ ^]^ Thus, we investigated whether endurance training mediated hepatic one‐carbon metabolism (**Figure** **5**a). Quantification of both PFC and serum samples revealed suppressed SAM levels under CRS, in addition to recovery in the exercise paradigms (Figure 5b,c). Liver *Mat1a* expression followed a similar pattern (Figure 5d), suggesting the possible involvement of hepatic metabolism. To provide more direct evidence, an AAV with liver‐specific promoter thyroxine binding globulin (TBG) was intravenously injected to drive the expression of shRNA targeting *Mat1a* (Figure 5e,f). The liver‐specific suppression of *Mat1a* in naïve animals (Extended Data Figure S5a–c, Supporting Information) attenuated circulating and central SAM levels as well as brain RNA methylation (Extended Data Figure S5d,f, Supporting Information), leading to depressed cortical activity (Extended Data Figure S5g, Supporting Information) and anxiety‐like behaviors (Extended Data Figure S5h–j, Supporting Information). These data suggest that normal hepatic biosynthesis of SAM is necessary for stress resilience. In CRS mice under exercise intervention, the downregulation of hepatic Mat1a (Figure 5g) remarkably suppressed circulating and brain SAM levels (Figure 5h,i), resulting in RNA demethylation in the PFC (Figure 5j). Consistent with previous data, we identified the inactivation of PrL neurons upon peripheral inhibition of SAM biosynthesis (Figure 5k–l). As a behavioral consequence, exercise‐conferred stress resilience was absent (Figure 5m–p), demonstrating the necessary role of exercise‐driven hepatic biosynthesis of methyl donors in the central regulation of anxiety‐like behaviors. Moreover, SAM may also work as the DNA methylation donor,^[^ ^38^ ^]^ and the DNA methylation such as 5‐methylcytosine (5mC) has been suggested in anxiety‐like behaviors.^[^ ^39^ ^]^ We further quantified 5mC concentration and relevant DNA methylase or inhibitors in mouse PFC but found no significant change under exercise paradigm or hepatic *Mat1a* knockdown (Extended Data Figure S6, Supporting Information). These results highlighted the role of liver biosynthesis of SAM in potentiating brain RNA m6A for conferring stress resilience.

**Figure 6:**
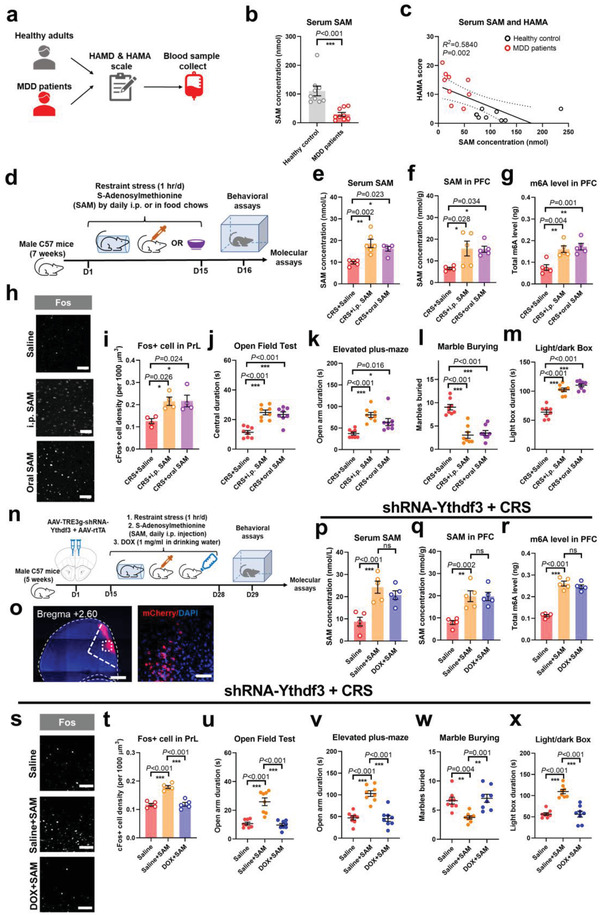
Circulating SAM is critical for anxiety disorders in both humans and rodents. a) Experimental schedules for a small‐cohort clinical study of serum SAM in major depressive disorder (MDD) patients. Both healthy controls and patients were interviewed using the Hamilton Depression Scale (HAMD) and Hamilton Anxiety Scale (HAMA) and then underwent serum SAM quantification. b) Compared to healthy controls, MDD patients had lower serum SAM levels. Two‐sample *t*‐test, *t*(16) = 4.435, *P* < 0.001. c) Serum SAM was inversely related to HAMA score, which reflected the severity of anxiety disorder. Linear regression, *R^2^* = 0.5840, *P* = 0.002. The dashed lines represent the 95% confidence interval (CI) of the regression curves. *N* = 9 individuals in each group. d) Schematic illustration of peripheral SAM replenishment. The RNA methylation donor, SAM, was introduced either via daily intraperitoneal injection (i.p., at 250 mg kg^−1^ daily) or via a customized, SAM‐enriched chow (0.77% in w/w based on AIN‐93 normal chows), in conjunction with CRS. Fourteen days later, behavioral tasks were performed. e) Peripheral supplementation of SAM remarkably elevated serum SAM level. One‐way ANOVA, *F*(2,13) = 10.38, *P* = 0.002. f) SAM replenishment elevated central SAM concentration *F*(2,12) = 5.789, *P* = 0.017. g) Brain m6A contents were also elevated with SAM administration *F*(2,12) = 13.08, *P* = 0.001. *N* = 5 mice in each group in e–g). h) Immunofluorescence staining of Fos+ cells in the PrL. Scale bar, 100 µm. i) Exogenous SAM significantly potentiated PrL neuronal activity in mice under CRS. *F*(2,9) = 7.004, *P* = 0.015; *N* = 4 mice (three slices each) from each group. j) Peripheral supplements of SAM remarkably relieved anxiety disorders under CRS as suggested by longer time spent in the central arena. *F*(2,21) = 21.93, *P* < 0.001. k) CRS mice with SAM intake spent more time in the open arm of the elevated plus‐maze. *F*(2,21) = 12.69, *P* < 0.001. l) SAM‐replenished mice presented less repetitive behaviors. *F*(2,21) = 30.71, *P* < 0.001. m) In CRS mice, SAM administration relieved light box avoidance behavior. *F*(2,21) = 61.41, *P* < 0.001. *N* = 8 mice in each group in j–m). n) Experimental timelines for SAM replenishment plus m6A‐reader inhibition assay. Mice were transfected with shRNA‐*Ythdf3* in the bilateral PrL 14 days before CRS plus SAM (i.p. injection), and DOX was added to the drinking water (1 mg/mL) to induce *Ythdf3* gene knockdown. o) Viral transfection site. Scale bar, 300 µm in left panel or 100 µm in right panel. p) While SAM supplementation in CRS mice increased the serum SAM level, the induction of *Ythdf3* gene knockdown did not affect peripheral SAM levels. Tukey's post‐hoc comparison following one‐way ANOVA (Saline+SAM vs DOX+SAM), *P* = 0.523. q) *Ythdf3* knockdown did not change central SAM levels. *P* = 0.998. r) The suppression of brain *Ythdf3* did not alter the local m6A concentration. *P* = 0.493. s) Immunofluorescent staining of Fos+ cells in the PrL. Scale bar, 100 µm. t) *Ythdf3* gene knockdown remarkably suppressed PrL neuronal activity in mice under CRS even with SAM injection. One‐way ANOVA, *F*(2,12) = 40.24, *P* < 0.001. *N* = 5 mice (three slices each) in each group. u) The improvement in time spent in central region following SAM injection in CRS mice was abolished by induced *Ythdf3* gene knockdown. *F*(2,21) = 27.05, *P* < 0.001. v) Blocking the *Ythdf3* gene also antagonized the anxiolytic effect of SAM as shown in the elevated plus‐maze task. *F*(2,21) = 29.06, *P* < 0.001. w) The repetitive marble burying behavior was reinstated under *Ythdf3* suppression. *F*(2,21) = 10.61, *P* < 0.001. x) *Ythdf3* gene knockdown abolished SAM's effect on relieving light avoidance behavior in CRS mice. *F*(2,21) = 33.73, *P* < 0.001. *N* = 8 mice in each group in u–x). ns, no significant difference, *P < 0.05, **P < 0.01, ***P < 0.001 using two‐sample *t*‐test in b) and using Tukey's post‐hoc comparisons in e–m, p–x). All data are presented as mean±SEM.

To generalize these rodent data to humans, we recruited a small group of patients with major depressive disorder (MDD) with prominent anxiety disorders (**Figure** **6**a). Compared to age‐ and sex‐matched healthy individuals (Extended Data Table S2, Supporting Information), patients displayed decreased circulating SAM levels (Figure 6b). Serum SAM levels were later found to be inversely correlated with the Hamilton Anxiety Scale (HAMA; Figure 6c). These data suggest the potential value of SAM as a biomarker for depression or anxiety disorders. We further tested the potency of SAM to mimic the effects of exercise on mouse models. Using intraperitoneal injection of SAM or feeding mice with SAM‐enriched food chow (Figure 6d), we successfully elevated both peripheral and central methyl donor levels (Figure 6e,f) and brain RNA methylation (Figure 6g). In CRS mice, peripheral supplementation of SAM‐activated PrL neurons (Figure 6h,i) to suppress anxiety‐like behaviors (Figure 6j–m). Furthermore, oral SAM intake elevated hepatic Mat1a and decreased brain *Alkbh5* gene expression (Extended Data Figure S7, Supporting Information), suggesting homeostatic regulation of body RNA methylation status. Finally, to validate the specificity of the SAM‐induced anxiolytic effect on brain m6A, we inhibited the expression of the m6A binding protein, *Ythdf3*, in the PrL of CRS mice receiving SAM injection (Figure 6n,o). Although the knockdown of *Ythdf3* did not change peripheral or central SAM levels (Figure 6p–q) nor brain m6A content (Figure 6r), it remarkably suppressed neuronal activity (Figure 6s,t). Consequently, the anxiolytic effect of SAM was antagonized by blocking the m6A binding pathway (Figure 6u–x). These data suggest that exogenous replenishment of methyl donors mimicked behavioral modulations of exercise training by improving brain m6A. Our data thus demonstrated a liver‐brain axis through which exercise facilitated brain RNA methylation to modulate prelimbic neural activity to improve stress resilience.

## 3 Discussions

To our knowledge, this is the first systemic study showing the involvement of brain RNA m6A in the physical exercise‐induced improvement of anxiety disorders. Previous studies have demonstrated the role of RNA methylation in the mPFC and hippocampus upon acute stress,^[^ ^11^ ^]^ and we further extended this observation to chronic environmental stress. More importantly, we established a hepatic mechanism in which liver biosynthesis of methyl donors was potentiated upon exercise training, leading to upregulated brain m6A that helped to activate cortical circuits for conferring stress resilience. Although exercise training has been suggested to mediate DNA methylation of specific genes,^[^ ^23^, ^24^ ^]^ our study provides a previously unrecognized role of mRNA methylation in exercise‐mediated anxiolysis.

Brain RNA m6A homeostasis is well orchestrated by various factors, of which *Mettl3* and *Mettl14* have been established to play roles in cognitive and mental functions.^[^ ^11^, ^25^, ^26^ ^]^ Regarding RNA demethylase, *Fto* mediates methylation status in psychiatric diseases^[^ ^40^ ^]^ or neurodegenerative disorders.^[^ ^41^ ^]^ Upon acute stress, *Fto* was slightly upregulated in the PFC,^[^ ^11^ ^]^ whereas we found no significant changes under CRS. Moreover, *Fto* has also been suggested to mediate N^6^,2'‐O‐dimethyladenosine (m6Am) of different RNA species,^[^ ^42^, ^43^ ^]^ although m6A is still attributed to be the main type of mRNA modification by *Fto*.^[^ ^44^ ^]^ Another demethylase, *Alkbh5*, presented potentiated expression in the CRS group, in agreement with previous findings in acute stress.^[^ ^11^ ^]^ In mouse depression models, ALKBH5 displayed nuclear redistribution to direct methylation of the fatty acid amide hydrolase (*FAAH*) gene.^[^ ^45^ ^]^ It is worth noting that a gene polymorphism of *Alkbh5* was previously found to be related to MDD risk in the Chinese Han population.^[^ ^46^ ^]^ These data support the role of RNA demethylases in mental functions. It is of equal importance to explore how environmental stress and exercise affect *Alkbh5* expression. A recent study reported that a circular noncoding RNA buffers *Alkbh5* function,^[^ ^45^ ^]^ providing an extra layer of epigenetic regulation. Here, we propose an alternative mechanism by suggesting that *Alkbh5* expression was inhibited upon oral intake of SAM (Extended Data Figure S7, Supporting Information). Therefore, the dynamic regulation of *Alkbh5* is closely related to methyl donor supply and/or brain m6A status, forming a homeostatic regulatory loop.

PrL neurons project to multiple cortical and subcortical regions to mediate emotional and motivational behaviors.^[^ ^47^ ^]^ Recent findings suggest the involvement of the cortico‐amygdala circuit in anxiety disorders.^[^ ^2^, ^32^, ^48^ ^]^ We recorded the in vivo activity of PrL‐BLA projecting neurons and found their suppression under CRS plus re‐elevation following the exercise intervention. Recent findings have shown that local infusion of ketamine activated cortical neurons and alleviated anxiety‐related behaviors.^[^ ^49^ ^]^ Given that both ketamine^[^ ^50^, ^51^ ^]^ and exercise^[^ ^17^ ^]^ can activate the mechanistic target of rapamycin (mTOR)‐directed spinogenesis and neural activities, physical exercise may have anxiolytic and antidepressant actions with long‐lasting effects. More importantly, our results support clinical observations showing the effectiveness of neural stimulation of the PFC in alleviating anxiety‐related behaviors.^[^ ^52^ ^]^ To provide the molecular substrate, we found that RNA demethylation of this PrL‐BLA circuit is sufficient to reverse exercise‐mediated effects, providing the first piece of evidence for the role of m6A within a specific neural circuit for mediating behavioral phenotypes.

From a clinical perspective, our human MDD cohorts displayed lower levels of circulating methyl donors, suggesting the possible involvement of RNA methylation in disease risk and pathogenesis. In recent decades, SAM has been employed in clinical trials against various psychiatric disorders, including MDD, although its overall efficiency needs to be further elaborated.^[^ ^53^, ^54^ ^]^ Mechanistic explanations for SAM in alleviating psychiatric disorders mainly relate to the regulation of folate and methionine metabolism in the biosynthesis of hormones or neurotransmitters,^[^ ^53^ ^]^ little attention has been paid to brain RNA methylation. As a universal methyl donor,^[^ ^55^ ^]^ SAM is known to mediate various biological macromolecules including DNA,^[^ ^56^ ^]^ histones^[^ ^57^ ^]^ and proteins,^[^ ^38^ ^]^ although its modulatory effect on brain RNA methylation remains poorly understood. Here, we provide results showing that circulating SAM can cross the blood–brain barrier to potentiate central m6A. Although SAM may also participate in other biological processes inside the brain, our data showing unchanged brain 5mC levels (Extended Data Figure S6, Supporting Information) revealed its critical role in RNA methylation. Moreover, we identified the effectiveness of intravenous injection or oral supplementation of SAM in preventing CRS‐induced anxiety‐like behaviors, similar to those of exercise effects. Thus, further studies should employ SAM as an early intervention strategy for subthreshold or high‐risk individuals with depression/anxiety disorders.

How exercise intervention elevates hepatic biosynthesis of SAM is an unresolved question. Methionine metabolism is a critical sensor that translates metabolic state into methylation process by generating SAM.^[^ ^58^ ^]^ Therefore, liver production of SAM is regulated by body metabolism status, which is prominently changed by persistent exercise. In particular, one‐carbon metabolism in the liver has been found to be potentiated by exercise training,^[^ ^59^ ^]^ supporting our results of enhanced SAM biosynthesis. When exploring the signaling pathway, it is worth noting that the mTOR pathway, which is a prominent cellular “metabolic sensor,” can fuel hepatic generation of SAM.^[^ ^60^ ^]^ Given the modulation of exercise on the liver mTOR pathway,^[^ ^61^, ^62^ ^]^ it is expected that endurance training will help augment SAM production within hepatic tissues. This working model may also help to explain anxiety and depression deficits in patients with liver dysfunction,^[^ ^63^, ^64^ ^]^ which remarkably impairs SAM biogenesis.^[^ ^35^ ^]^ Due to the lack of studies illustrating the causal relationship between body metabolism and brain epigenetic regulation, our study provides novel insights for understanding brain adaptations upon exercise training from the perspective of the metabolic‐epigenetic axis.

The current work has established the necessity of brain RNA m6A under exercise‐mediated anxiolytic effects, as supported by the assay to depress cortical m6A levels (Figure 2k–t) or to disrupt the m6A‐dependent translational process (Figure 6s–x; and Extended Data Figure S2j–o, Supporting Information). Our results agree with previous findings showing the role of endurance training in alleviating anxiety disorders,^[^ ^12^, ^13^ ^]^ and provides extra layers of epigenetic regulation by exercise training in addition to DNA or histone modification.^[^ ^20^, ^21^, ^23^ ^]^ On the other hand, the illustration of this m6A pathway also implies the possible limitation of exercise intervention. For example, the dependence on hepatic‐brain axis suggests the ineffectiveness of exercise training on people with hepatic dysfunctions which may interfere with normal SAM biosynthesis. Moreover, the exercise‐induced anxiolysis might be potentiated by further replenishment of RNA methylation donors, providing a strategy of exercise plus diet supplement in preventing anxiety disorders.

In summary, a liver‐brain pathway exists to mediate the liver biosynthesis of methyl donors, which can affect RNA methylation status in mPFC neurons. CRS disrupts metabolic regulation of brain epigenetic homeostasis to impair cortical activity, and exercise remarkably restores cortical RNA m6A levels by enhancing hepatic biosynthesis, contributing to improved resilience to counteract environmental stress. Our current studies demonstrated a previously unrevealed pathway by which exercise prevents stress‐induced anxiety disorders and may benefit the further development of complementary treatments for mental health.

## Conflict of Interest

The authors declare no conflict of interest.

## Author Contributions

All experiments were designed by L.Z. L.Y. performed molecular, histological, and behavioral assays. Y.J. and X.L. recruited the patients and collected blood samples. J.W. performed the two‐photon calcium imaging and data analysis. F.Y., T.C., M.W., and S.W. assisted in the molecular studies, histological experiments, and behavioral phenotyping assays. The manuscript was written by L.Z. and L.Y. with help and comments from all authors, and was approved by K.‐F.S. and L.Z. The experiment was cosupervised by Y.J., K‐F.S., and L.Z.

## Supporting information

Supporting Information

## Data Availability

The data that support the findings of this study are available from the corresponding author upon reasonable request.
