# Supplementary material for: Physical Exercise Prevented Stress‐Induced Anxiety via Improving Brain RNA Methylation
Source: Adv Sci (Weinh). 2022 Jun 1;9(24):2105731. doi: 10.1002/advs.202105731 (PMC9404392; doi:10.1002/advs.202105731)
Supplement: Supplementary file 1 — Supporting Information [file ADVS-9-2105731-s001.pdf]

ADVANCED SCIENCE
Open Access

Supporting Information

for *Adv. Sci.*, DOI 10.1002/advs.202105731

Physical Exercise Prevented Stress-Induced Anxiety via Improving Brain RNA Methylation

Lan Yan, Ji-an Wei, Fengzhen Yang, Mei Wang, Siqi Wang, Tong Cheng, Xuanjun Liu, Yanbin Jia, Kwok-Fai So* and Li Zhang**

Supplemental Information

Physical exercise prevented stress-induced anxiety via improving brain RNA methylation

This supplemental file includes:

Methods and Materials

Extended Data Figures 1-7

Extended Data Tables 2-4

(Extended Data Table 1 is attached separately as an Excel file)

Methods and Materials

Experimental animals

Male C57BL/6J mice (5 weeks old) were purchased from Guangdong Medical Laboratory Animal Center and were used for all experiments. All animals were group-housed under normal light-dark cycle (0700-1900 on light) with food and water *ad libitum*. The exact *n* number for each experiment was specified in relevant result or figure legend sections. All animal experimental protocols were pre-approved by the Ethics Committee of Experimental Animals of Jinan University in accordance with Institutional Animal Care and Use Committee guidelines for animal research.

For chronic stress restraint (CRS) model, each mouse was put into a small tube (10 cm length, and 3.5 cm diameter, with punctured holes in the head end for ventilation) for 1 hr (7 pm to 8 pm, or ZT 1200-1300) daily, within 14 consecutive days. In the exercise paradigm, the animals were put on a treadmill apparatus (Zhongshi Tech., China) from 9 to 10 am (ZT 0200-0300) or 9-10 pm (ZT 1400-1500) for 14 consecutive days. The velocity was maintained at 10 m/min.

Behavioral tests

Open field test: The open field test (OFT) was carried out in a plastic chamber (40 × 40 × 30 cm) with light overhead. An arbitrary central region was defined with 20 × 20 cm size. At the beginning of the experiment, one mouse was gently placed on the center and allowed to freely explore the arena for 5 min. Movement paths were captured by an infrared camera and analyzed using EthoVision ver 7.0 (Noldus, Netherland).

Elevated plus-maze: The elevated plus-maze (EPM) was initiated by putting the mouse into the central platform (5 × 5 cm) of the elevated plus maze. The animal was then allowed freely explore the arena consisting of two opposing open arms (30 × 5 × 0.5 cm) and two opposing enclosed arms (30 × 5 × 15 cm) within 5 min. The duration time in the open and closed arms was analyzed by EthoVision package.

Marble burying test: The test animal was firstly allowed to explore the test cage (35 × 20 × 10 cm) for 10 min as the habituation. The cage was then filled with approximately 5 cm layer of husk bedding materials that were evenly distributed with a total of 15 glass marbles (1.4 cm in diameter, plain dark glass, spaced evenly in a 3 × 5 grid on the surface of the bedding). The test phase started by placing the mouse into the cage and allowed it to explore it for 10 min. At the end of the test, mice were removed from the cage and the number of marbles that were buried with bedding up to 2/3 of their depth was counted. Each mouse was tested for three times and the average number was calculated. The counting was performed by the same personnel who was blinded to the group.

Light/dark box test: Two boxes are (16 × 16 × 25cm) were connected by a small hole, through which the mouse can freely shuttle between two chambers. The dark box was painted in black and covered with a black lid, and the light box was painted in white and illuminated by a 60 W light bulb. At the beginning of the experiment, the mouse was put in the middle of the white box. The duration time in the light and dark box during single 5-min recording period was analyzed by EthoVision package.

All behavioral tests were performed during the dark phase (1900-2200), and the light intensity was maintained at 100~200 lux, except for the light box.

Serum extraction from human blood samples

The blood samples were obtained from patients with depression (3 males and 6 females) and healthy individuals (4 males and 5 females), with no known exposure to anti-psychotic or anti-inflammatory drugs within 3 months. All subjects aged between 18 and 40 years. Clinical diagnosis of depression was established according to a DSM-5 criteria (2013). Healthy control subjects were age-matched with patient group and has no history of mental disorder or major systemic diseases.

Plasma was prepared from venous blood collected in EDTA-containing tubes, and was then centrifuged at 1000 g. The serum was collected and stored at -80ºC until use. Before anticipation, EDTA was removed using a 3.5 kDa D catheter dialyzer plasma (Millipore, US) in PBS. An ethical approval for this study has been obtained from the Research Ethics Board of Research Ethics Committee of the First Affiliated Hospital of Jinan University (Guangzhou, China).

Real-time qPCR

Total RNA was isolated using Trizol Reagent (Invitrogen, USA). After mRNA quantification, equal amounts of cDNA were synthesized using the High-Capacity cDNA Reverse Transcription kit (TaKaRa, Japan). Real-time qPCR was performed in TB Green Premix Ex Taq (TaKaRa) and specific primers, using GAPDH as the internal control. qPCR was carried out in a CFX384 Real Time System (Bio-Rad, USA). Primer sequences are listed in Extended Data Table 3.

Western blotting

Tissue lysates were extracted from the mouse PFC in radioimmunoprecipitation assay buffer containing protease and phosphatase inhibitors. After quantification using bicinchoninic acid kit (Beyotime, China), 10 μg protein samples were separated by SDS–polyacrylamide gel electrophoresis under 120 V for 60 min and were transferred to the polyvinylidene fluoride membrane at 300 mA for 90 min. After washing in phosphate-buffered saline (PBS) with Tween 20 and blocking in 5% bovine serum albumin (BSA), the membrane was incubated with the primary antibody at 4°C overnight. The membrane was washed and incubated in secondary antibody for 2 hours. Protein bands were visualized using an imaging system (Bio-Rad, USA). Integrated gray values of each band were measured using ImageJ (National Institutes of Health, Bethesda, USA). The full list of primary and secondary antibody used was shown in Extended Data Table 4.

Immunofluorescence staining

Mice were euthanized using isoflurane. After infusion of saline and paraformaldehyde (PFA), the whole brain was extracted and dehydrated with 30% sucrose overnight. Following fixation in PFA, the brain tissue was sectioned into 40 μm coronal slices using a sliding microtome (Leica, Germany). After PBS washing and blocking, the brain slices were incubated with primary antibody at 4°C for 48 hours, followed by secondary antibody. Images were captured with a confocal microscope (LSM700, Zeiss, Germany), and the fluorescent intensity was analyzed using ImageJ. The full list of primary and secondary antibody used was shown in Extended Data Table 4.

In quantification of immunofluorescent staining such as cFos, 3 slices (20 μm thickness) were sampled from each mouse, with 40 μm interval. The number of cFos+ cells in each slice was normalized against the total volume of the tissue region, and the cell density was averaged to get the number of each individual animals. In plotting the figures, each dot represented the averaged value from one animal.

Enzyme linked immunosorbent assay (ELISA)

The quantification of m6A, m5C and SAM was performed in mPFC and serum samples using ELISA kits (m6A: Epigentek, USA; m5C: Abcam, USA; SAM: Biovision, China) following the manufacturer's instructions.

Intravenous infusion of virus

A total of 200 μl diluted AAV2/8 viral vectors (dosage: 5×10^{12} vg/kg) containing TBG promoter driving the expression of shRNA targeting Mat1a (GenBank accession: NM_11720) were administrated via the tail vein.

Stereotactic injection of virus

Four-week old C57BL/6 mice were anesthetized with 1.25% Avertin. After making an incision on the scalp and local sterilization, the periosteum tissue was removed. The injection site was located under the help of stereotactic apparatus (RWD, China) using coordinates (PrL: 2.65 mm anterior, ±0.2 mm against the midline, and 0.9 mm depth below the dura; BLA: -1.40 mm posterior of Bregma, ±3.1 mm lateral, and 4.5 mm depth). The viral titer was maintained at around 5×10^{12}

gene copies (GC) per ml, and a total of 0.2 μl viral solution was introduced for each site. Viral injection was performed using a glass micropipette connecting to a computerized ultra-micro injection pump (Nanoliter 2010, WPI, USA). The micropipette was retained for 10 min before retraction. The full list of viral vectors used was shown in Extended Data Table 4. Following experimental procedures usually initiated for more than 14 days later.

***In vivo* calcium imaging**

Following established protocols [1], the mice were anesthetized with 1.25% Avertin, and the scalp was incised to expose the skull. After leaning the surface of skull, two metal bars were glued to the rostral and caudal skull. Dental cement was then applied surrounding the imaging region to fix the metal bars. At 24 hr later, an imaging window (2 mm by 2 mm) around the medial prefrontal cortex (mPFC, 4.60 mm anterior and 0.40 mm lateral) was created by a high-speed micro-drill. The exposed brain matter was cover by a glass coverslip using Vetbond Tissue Adhesive (3M, USA). The mouse was then fixed on the stage of 2-photon microscope (LSM780, Zeiss, Germany). An imaging session was performed using 920 nm excitation laser with a water-immersed objective (20×, 1.1 numerical aperture; ZEISS, Germany). *In vivo* calcium recording was performed as previously described [2]. Briefly speaking, the calcium activities of layer I/II somas (80 to 250 um in depth) of pyramidal neurons were recorded at 2 Hz during a 2.5-min period. The laser power was restricted below 25 mW during the data acquisition. Acquired time series images were corrected by TurboReg module of ImageJ. The fluorescent value F was quantified by average pixels extracted from designed region of interest covering identifiable soma. The $\Delta F/F_0$ was calculated as $(F-F_0)/F_0$, where the F_0 was averaged F values during the first 10% recording period

as the basal level. A calcium transient was defined when the $\Delta F/F_0$ is higher than three folds of sample standard deviations (SD). For the presentation of heatmap, $\Delta F/F_0$ was further translated into z-score, which was defined as the number of sem below/above the sample average level (z score = (sample value – average value) / sem of sample value).

m6A dot blot assay

Total RNA was extracted from isolated PFC tissues using Trizol reagent (Invitrogen, US) and Direct-zol RNA MiniPrep kit (Zymo Research, UK) following the manual instruction. For the dot blot assay, RNA was firstly denatured at 65 °C for 5 min, and was serially diluted into 600, 300 and 150 ng/μl. Samples were then loaded onto an Amersham Hybond-N+ membrane (GE Healthcare, USA) in duplicates. The membrane was UV crosslinked for 5 min and washed by PBST. The Methylene blue (Sangon Biotech, China) was used to reveal the total RNA amounts. After blocking in 5% BSA, the membrane was incubated with specific m6A antibody (1:1000, Synaptic System, Germany) at 4 °C overnight. On the next day, HRP-conjugated anti-mouse IgG was added for 1-hr incubation, followed by development and imaging capture in an automated system (Bio-Rad, US).

Immunoprecipitation (IP)-based m6A-seq and bioinformatics analysis

In m6A-seq assay, PFC tissues from 3 mice were pooled together to generate one biological sample, and 3 biological replicates were enrolled in each group. Total RNA was extracted from isolated PFC tissues using Trizol reagent (Invitrogen, US) and Direct-zol RNA MiniPrep kit (Zymo Research, UK) following the manual instruction. The total RNA quality and quantity were

analyzed by Bioanalyzer 2100 and RNA 6000 Nano LabChip Kit (Agilent, USA) with RIN number >7.0. Approximately more than 25 μg of total RNA was used to deplete ribosomal RNA according to the manuscript of the Epicentre Ribo-Zero Gold Kit (Illumina, USA). The ribosomal-depleted RNA was then fragmented into ~100 nt length oligonucleotides using divalent cations under elevated temperature. The cleaved RNA fragments were subjected to incubation for 2 hr at 4 ℃ with m6A-specific antibody (No. 202003, Synaptic Systems, Germany) in IP buffer (50 mM Tris-HCl, 750 mM NaCl and 0.5% Igepal CA-630) supplemented with BSA (0.5 μg μl^{-1}). The mixture was then incubated with protein-A beads and eluted with elution buffer (1 × IP buffer and 6.7mM m6A). Eluted RNA was precipitated by 75% ethanol. Eluted m6A-containing fragments (IP) and untreated input control fragments were converted to final cDNA library in accordance with a strand-specific library preparation by dUTP method. The average insert size for the paired-end libraries was ~100±50 bp. RNA sequencing was performed based on the paired-end 2×150bp sequencing on an Illumina Novaseq™ 6000 platform at the LC-BIO Bio-tech ltd (Hangzhou, China) following the vendor's recommended protocols.

Cutadapt [3] and perl scripts in house were used to remove the reads that contained adaptor contamination, low quality bases and undetermined bases. Then sequence quality was verified using FastQC (http://www.bioinformatics.babraham.ac.uk/projects/fastqc/). We used HISAT2 [4] to map reads to the genome of Mus musculus (ver 96) with default parameters. Mapped reads of IP and input libraries were provided for R package exomePeak [5], which identifies m6A peaks with bed or bam format that can be adapted for visualization on the UCSC genome browser or IGV software (http://www.igv.org/). MEME [6] and HOMER [7] were used for de novo and known motif finding followed by localization of the motif with respect to peak summit by perl

scripts in house. Called peaks were annotated by intersection with gene architecture using ChIPseeker [8]. StringTie [9] was finally used to perform expression level for all mRNAs from input libraries by calculating FPKM (=[total_exon_fragments / mapped_reads(millions) × exon_length(kB)]). The differentially expressed mRNAs were identified with log2 (fold change) >1 or log2 (fold change) <-1 and *P* value < 0.05 by R package edgeR [10]. The FDR correction was used to calculate the corrected *P* value.

Statistical Analysis

All data were not transformed and presented in their original format with no exclusion. The only exception is the processing of calcium recording data, which were normalized against their basal levels (see ***In vivo* calcium imaging** section for details). All data were presented as mean ± standard error of means (sem). Each dataset was firstly tested for normal distribution. Those fitted Gaussian distribution were enrolled into parametric statistical tests. In specific, two-sample student *t*-test was used to compare two samples, whilst one-way analysis of variance (ANOVA) was adopted for the comparison among multiple groups, followed by Tukey's post hoc test in comparing two groups. For two independent variables, two-way ANOVA and Tukey's post hoc comparison were adopted. For those data not fitted with normal distribution, nonparametric Kolmogorov-Smirnov test was used under 2-group comparison, and Kruskal-Wallis test was adopted for multi-group comparison, followed by Dunn's post hoc comparisons. Two-sided test was used under all cases, and a significant level was defined when P<0.05. The sample size (*N*) was stated as in the figure legends. All statistical analysis was performed by GraphPad Prism ver 8.0 (GraphPad Inc, USA).

References:

1. Chen, K., et al., *Exercise training improves motor skill learning via selective activation of mTOR.* Sci Adv, 2019. **5**(7): p. eaaw1888.
2. Holtmaat, A., et al., *Long-term, high-resolution imaging in the mouse neocortex through a chronic cranial window.* Nat Protoc, 2009. **4**(8): p. 1128-44.
3. Kechin, A., et al., *cutPrimers: A New Tool for Accurate Cutting of Primers from Reads of Targeted Next Generation Sequencing.* J Comput Biol, 2017. **24**(11): p. 1138-1143.
4. Kim, D., B. Langmead, and S.L. Salzberg, *HISAT: a fast spliced aligner with low memory requirements.* Nat Methods, 2015. **12**(4): p. 357-60.
5. Meng, J., et al., *A protocol for RNA methylation differential analysis with MeRIP-Seq data and exomePeak R/Bioconductor package.* Methods, 2014. **69**(3): p. 274-81.
6. Bailey, T.L., et al., *MEME SUITE: tools for motif discovery and searching.* Nucleic Acids Res, 2009. **37**(Web Server issue): p. W202-8.
7. Heinz, S., et al., *Simple combinations of lineage-determining transcription factors prime cis-regulatory elements required for macrophage and B cell identities.* Mol Cell, 2010. **38**(4): p. 576-89.
8. Yu, G., L.G. Wang, and Q.Y. He, *ChIPseeker: an R/Bioconductor package for ChIP peak annotation, comparison and visualization.* Bioinformatics, 2015. **31**(14): p. 2382-3.
9. Pertea, M., et al., *StringTie enables improved reconstruction of a transcriptome from RNA-seq reads.* Nat Biotechnol, 2015. **33**(3): p. 290-5.
10. Robinson, M.D., D.J. McCarthy, and G.K. Smyth, *edgeR: a Bioconductor package for differential expression analysis of digital gene expression data.* Bioinformatics, 2010. **26**(1): p. 139-40.

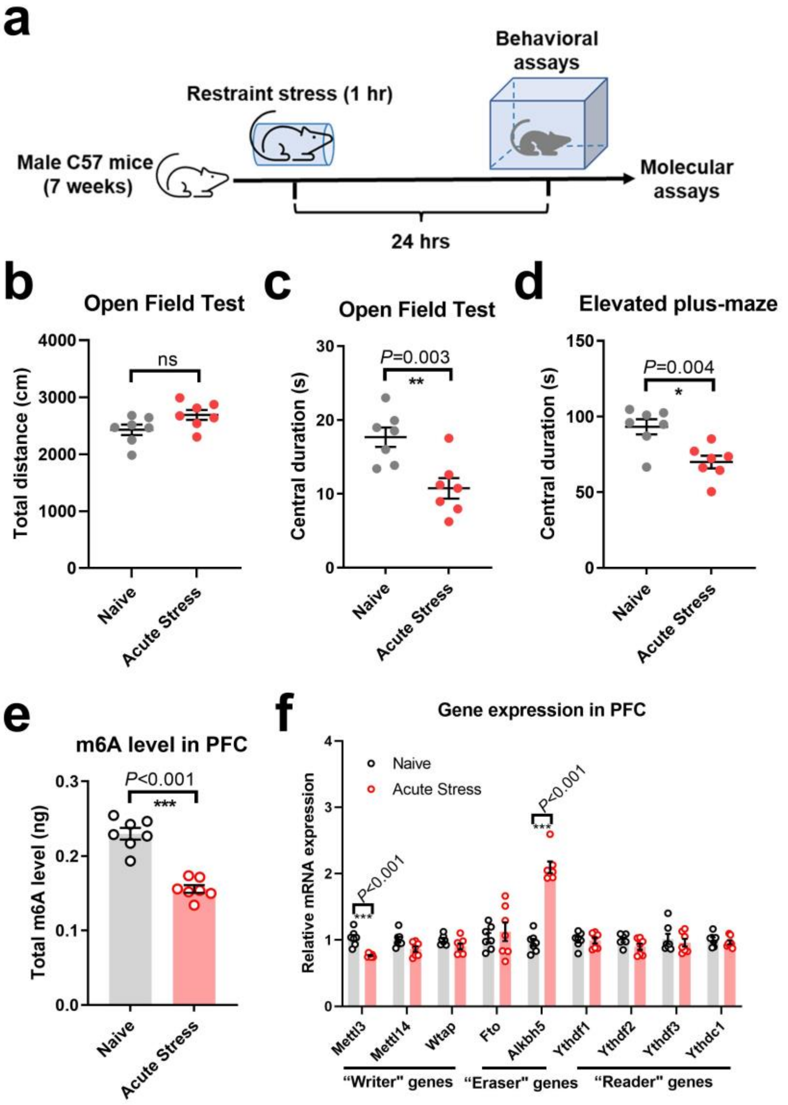

Extended Data Figure 1. Acute restraint stress suppresses brain m6A. (a) Experimental protocols. Male C57BL/6 mice were treated under restraint stress for 1 hr, followed by behavioral assays at 24 hr later. **(b)** Mice under environmental stress presented unchanged motor activity. Two-sampled *t*-test, $t(12)=2.077$, $P=0.060$. **(c)** Acute stress induced lower central durations. Two-sampled *t*-test, $t(12)=3.631$, $P=0.003$. **(d)** Mice in stress group also showed lower duration in the open arm during the elevated plus-maze task. Two-sampled *t*-test, $t(12)=3.597$, $P=0.004$. **(e)** Acute stress suppressed m6A levels in PFC. Two-sampled *t*-test, $t(12)=7.951$, $P<0.001$. **(f)** Relative expression levels of RNA methylation regulator genes. Acute stress inhibited expression

of *Mettl3* gene and increased the expression of RNA demethylase gene *Alkbh5* (P<0.001 in both genes under multiple *t*-test) whilst other genes had unchanged expression. N=7 mice in each group in **(b-f)**. ns, no significant change; *P<0.05, **P<0.01, ***P<0.001. All data are presented as mean±SEM.

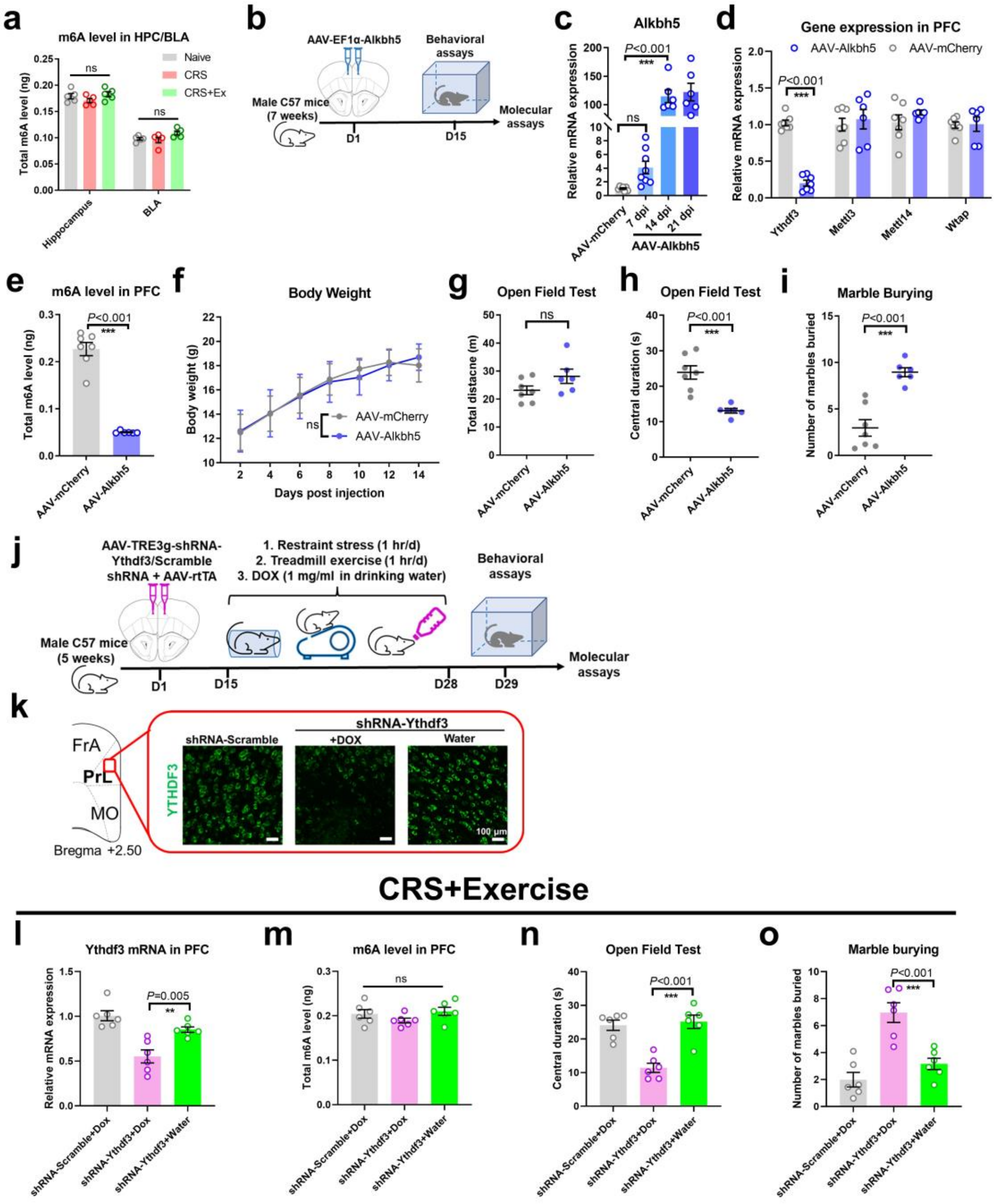

Extended Data Figure 2. Brain RNA methylation mediates stress resilience. (a) Total m6A levels in hippocampus or basolateral amygdala (BLA) were not changed by CRS or exercise training. Multiple *t*-test, $P>0.05$ in both cases. **(b)** Experimental protocols for Alkbh5-mediated RNA demethylation in generating anxiety-like behaviors. **(c)** Adeno-associated virus (AAV)-mediated transfection of Alkbh5 gene resulted in gradual increased gene expression level from day 7 post injection (7 dpi), and reached the peak value since 14 dpi. One-way ANOVA,

F(3,23)=63.01, *P*<0.001. *N*=7, 8, 6 and 6 mice in AAV-mCherry, 7 dpi, 14 dpi and 21 dpi groups, respectively. **(d)** Bilateral transfection of AAV-Alkbh5 remarkably increased gene expression of Ythdf3. Multiple *t*-test, *P*<0.001. **(e)** Alkbh5 over-expression decreased brain m6A levels in naïve mice. Two-sample *t*-test, *t*(11)=11.67, *P*<0.001. **(f)** Unchanged body weight by central down-regulation of m6A. Two-way ANOVA with respect to group×time factor, *F*(6,140)=0.0395, *P*=0.997. **(g)** Suppressing brain m6A level did not change total distance in the open field. Two-sample *t*-test, *t*(11)=1.473, *P*=0.109. **(h)** Alkbh5 over-expression suppressed time spent in the central region. Two-sample *t*-test, *t*(11)=5.039, *P*<0.001. **(i)** RNA demethylation in mPFC induced more repetitive behaviors. Two-sample *t*-test, *t*(11)=5.672, *P*<0.001. *N*=7 and 6 in mCherry and Alkbh5 groups, respectively, in **(d-i)**. **(j)** Experimental timelines for RNA binding protein Ythdf3 in anxiety behaviors. Mice received bilateral injection of AAV carrying shRNA of Ythdf3 under the direction of TRE3g promoter, in conjunction of a second AAV expressing transcription factor rtTA. By 14-day oral infusion of doxycycline (Dox) to activate rtTA driving shRNA-Ythdf3 in conjunction with CRS and treadmill exercise, behavioral tests were performed. **(k)** Immunofluorescent staining of Ythdf3 expression in prelimbic (PrL) region. FrA, frontal association cortex; MO, medial orbitofrontal cortex. Scale bar, 100 μm. **(l)** Relative gene expression of Ythdf3 was depressed by shRNA. One-way ANOVA, *F*(2,15)=17.11, *P*<0.001. **(m)** Down-regulation of Ythdf3 did not affect the total m6A level. One-way ANOVA, *F*(2,15)=1.699, *P*=0.216. **(n)** Ythdf3 knockdown decreased central duration in the open field. One-way ANOVA, *F*(2,15)=21.42, *P*<0.001. **(o)** Gene knockdown of Ythdf3 also induced more repetitive behaviors in marble burying paradigms. One-way ANOVA, *F*(2,15)=20.22, *P*<0.001. *N*=6 mice in each group in **(l-o)**. ns, no significant difference; **P*<0.05, ***P*<0.01, ****P*<0.001 using Tukey

post-hoc comparison in **(c, l-o)**, or using 2-sample unpaired *t*-test in **(e-i)**. All data are presented as mean±SEM.

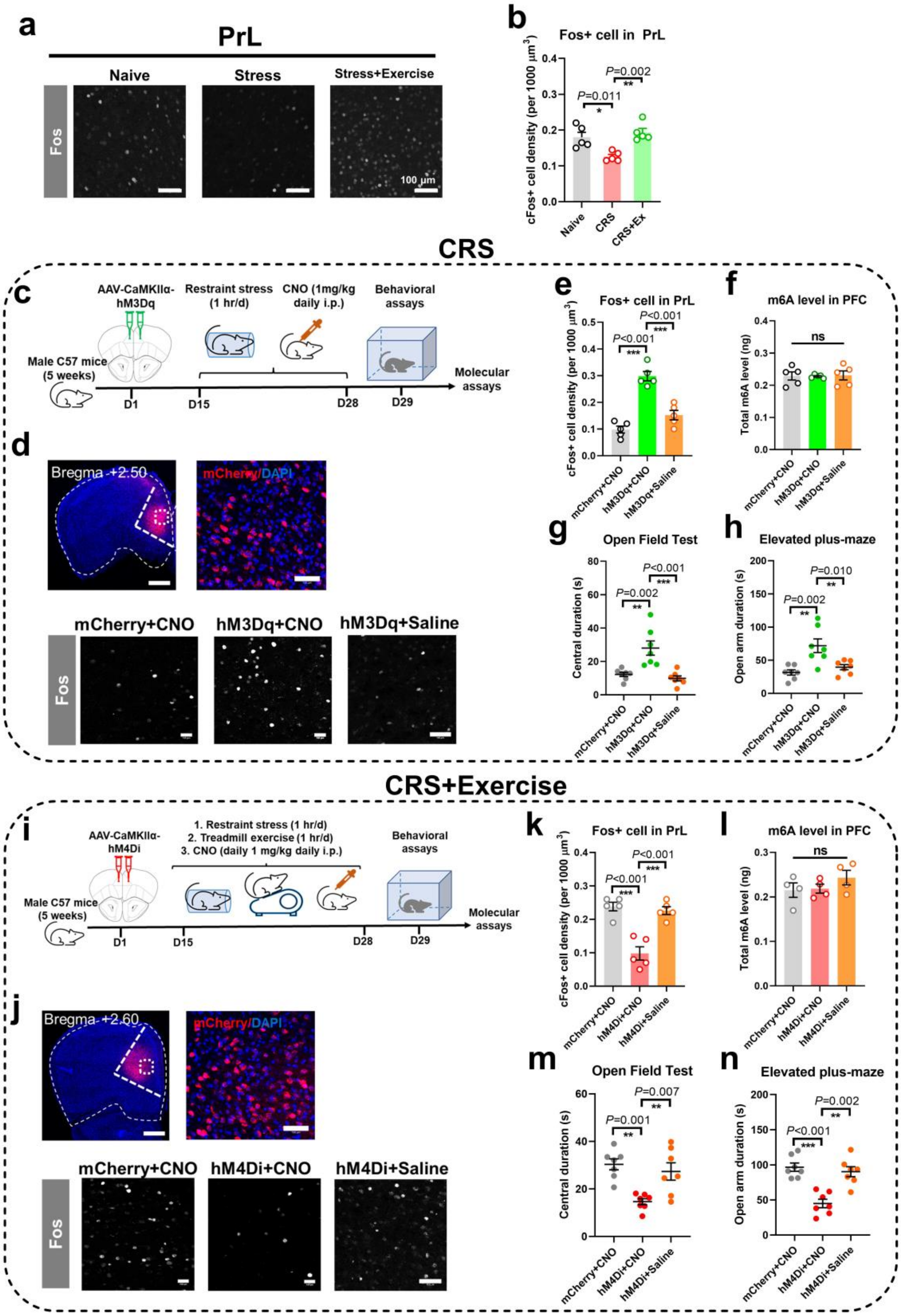

Extended Data Figure 3. Exercise activates PrL neurons to confer stress resilience. (a) Immunofluorescent staining of Fos expression in PrL region. Scale bar, 100 μm. **(b)**

Quantification of Fos+ cells in PrL slice. One-way ANOVA, $F(2,12)=10.86$, $P=0.002$. $N=5$ mice in each group (3 slices from each mouse). **(c)** Experimental protocols for the sufficiency of PrL activation in attenuating environmental stress. CRS paradigm was initiated at 14 days after introducing hM3Dq receptor into bilateral PrL, in conjunction with daily infusion of clozapine-N-oxide (CNO, at 1mg/kg via i.p. injection). **(d)** Immunofluorescent staining of viral infection site (upper panels) and Fos activity in PrL (lower panels). Scale bar, 300 μm in panoramic view or 100 μm in enlarged images. **(e)** Quantification of Fos+ cells. CNO infusion effectively elevated neuronal activity with hM3Dq transfection. $F(2,12)=41.07$, $P<0.001$. $N=5$ mice in each group (3 slices from each mouse). **(f)** Unchanged m6A contents in PFC extracts by chemogenetic activation. $F(2,12)=0.0177$, $P=0.983$. $N=5$ mice in each group. **(g)** PrL activation increased time spent in the central arena $F(2,18)=13.18$, $P<0.001$. (**h**) Chemogenetic activation of PrL increased open arm duration on the elevated plus-maze. $F(2,18)=9.928$, $P=0.001$. $N=7$ mice in each group in **(g-h)**. **(i)** Schematic illustrations for the necessity of PrL activation in exercise-conferred stress resilience. CRS and exercise paradigm started at 14 days after transfecting hM4Di receptor into bilateral PrL, in parallel with daily infusion of CNO. **(j)** Immunofluorescent staining of viral transfection and Fos activity in PrL. Scale bar, 300 μm in panoramic view or 100 μm in enlarged images. **(k)** Quantification of Fos+ cells. Chemogenetic inhibition suppressed neuronal activity. $F(2,12)=26.03$, $P<0.001$. $N=5$ mice in each group (3 slices from each mouse). **(l)** Unchanged m6A contents upon chemogenetic inhibition. $F(2,9)=1.109$, $P=0.371$. $N=4$ mice each group. **(m)** PrL inhibition abolished exercise-mediated anxiolytic effects as suggested by lower central time. $F(2,18)=10.33$, $P<0.001$. (**n**) Animals exhibited shorter open arm preference after PrL inhibition. $F(2,18)=19.57$, $P<0.001$. $N=7$ mice in each group in **(m-n)**. ns,

no significant difference; *P<0.05, **P<0.01, ***P<0.001 using Tukey post-hoc comparison in **(b, e-h, k-n)**. All data are presented as mean±SEM.

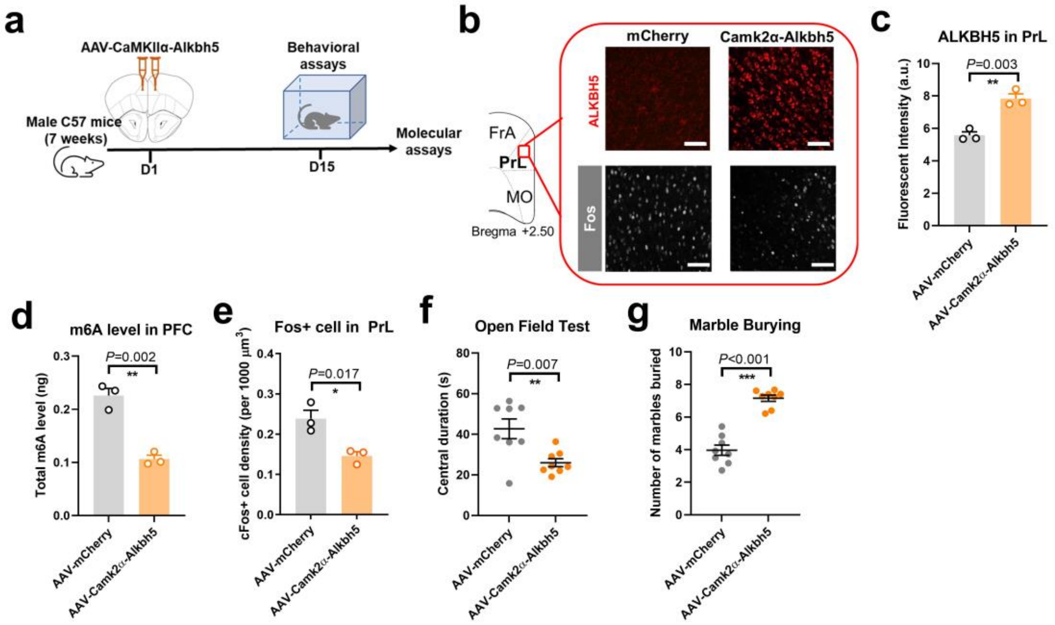

Extended Data Figure 4. RNA methylation in cortical excitatory neurons is necessary for maintaining stress resilience. (a) Experimental schedules, in which mice received behavioral tests at 14 days after transfecting Alkbh5 gene into excitatory neurons of PrL. **(b)** Immunofluorescent staining of Alkbh5 (upper panels) and Fos activity (lower panels) in PrL. Scale bar, Scale bar, 100 µm. **(c)** Quantification of Alkbh5 immunoreactivity, as presented in arbitrary unit (a.u.) of fluorescent intensity. Two-sample *t*-test, *t*(4)=6.289, *P*=0.003. **(d)** Downregulation of brain m6A after over-expressing Alkbh5. Two-sample *t*-test, *t*(4)=7.754, *P*=0.002. **(e)** Suppressed cortical activity by over-expressing Alkbh5. Two-sample *t*-test, *t*(4)=3.959, *P*=0.017. *N*=3 mice in each group in **(c-e)**. **(f)** Suppressing RNA methylation in excitatory neurons shortened duration in central field. Two-sample *t*-test, *t*(14)=3.184, *P*=0.007; **(g)** and induce more marble burying behaviors. Two-sample *t*-test, *t*(14)=8.644, *P*<0.001. *N*= 8 mice each group in **(f-g)**. All data are presented as mean±SEM.

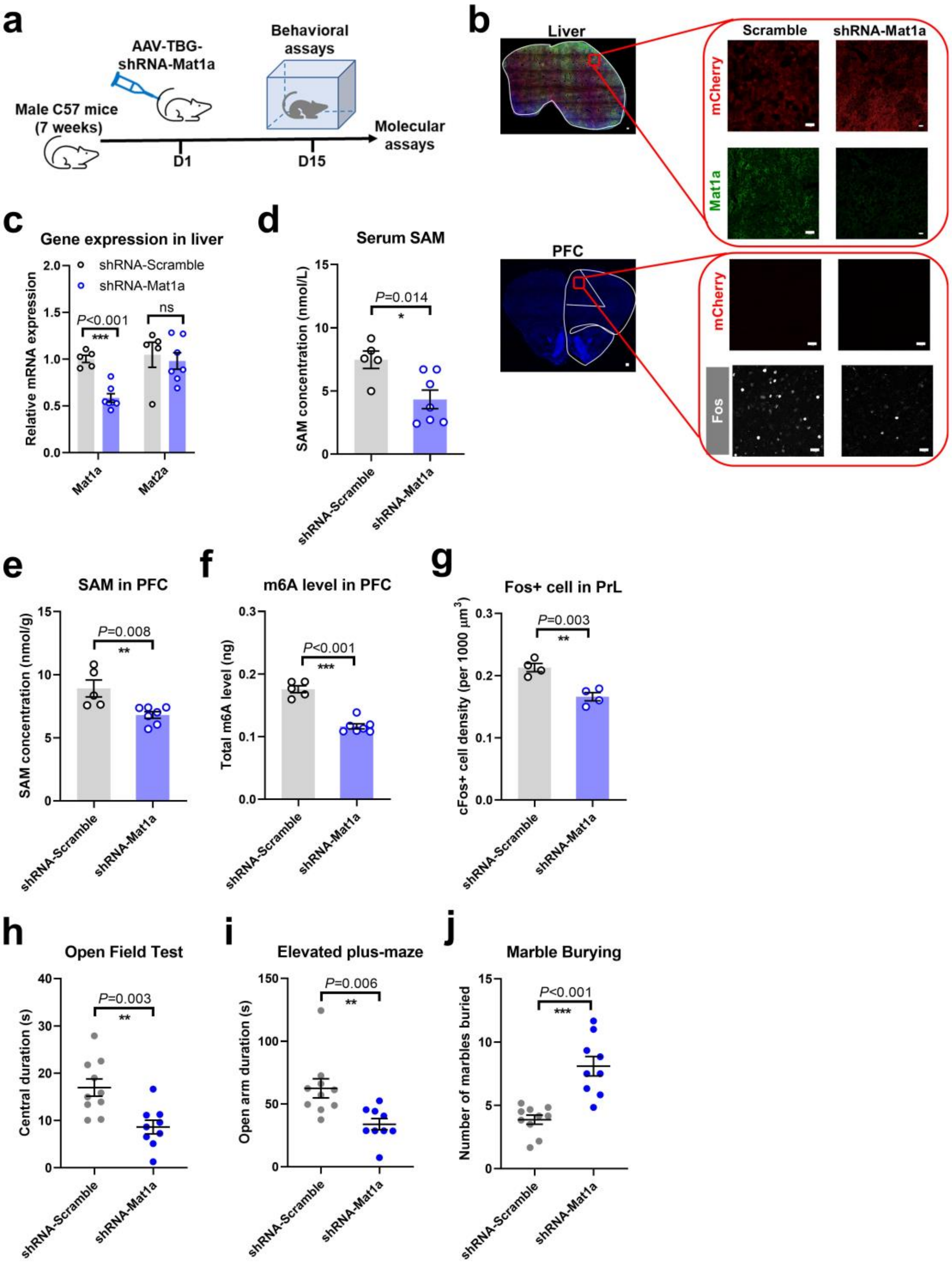

Extended Data Figure 5. Hepatic biosynthesis of methyl donor is necessary for preventing anxiety disorders. (a) Schematic illustration of liver-specific down-regulation of SAM biosynthesis. Liver-targeted AAV vector containing shRNA of Mat1a was administrated into naïve mice via intravenous injection, followed by behavioral phenotyping after 14 days. **(b)** Upper

panels: Immunofluorescent imaging of liver slices showed suppression of Mat1a expression. Lower panels: PFC slices demonstrating minimal viral transfection in the brain, as well as Fos activity under Mat1a down-regulation in liver. Scale bar, 50 μm. **(c)** Down-regulation of Mat1a (P<0.001) but not Mat2a gene (P=0.673) in hepatic tissues after shRNA transfection. ns, no significant difference; N=5 and 7 mice in Scramble and shRNA-Mat1a groups, respectively. **(d-f)** Liver-specific down-regulation of Mat1a decreased circulating SAM level (Two-sample *t*-test, $t(10)$=2,985, P=0.014; **d**), brain SAM levels (Two-sample *t*-test, $t(10)$=3.302, P=0.008; **e**) and central m6A concentration (Two-sample *t*-test, $t(10)$=8.906, P<0.001; **f**). N=5 and 7 mice in Scramble and shRNA-Mat1a groups, respectively. **(g)** Mat1a knockdown significantly suppressed PrL activity. Two-sample *t*-test, $t(6)$=4.974, P=0.003. N=4 mice in each group. **(h-j)** Hepatic-specific Mat1a silencing induced anxiety-like behaviors in naïve mice as suggested by lower central time in the open field arena (Two-sample *t*-test, $t(17)$=3.538, P=0.003; **h**), shorter open arm duration in elevated plus-maze (Two-sample *t*-test, $t(17)$=3.148, P=0.006; **i**) and more frequent marble burying behaviors (Two-sample *t*-test, $t(17)$=5.134, P<0.001; **j**). N=10 and 9 mice in Scramble and shRNA-Mat1a groups, respectively. All data are presented as mean±SEM.

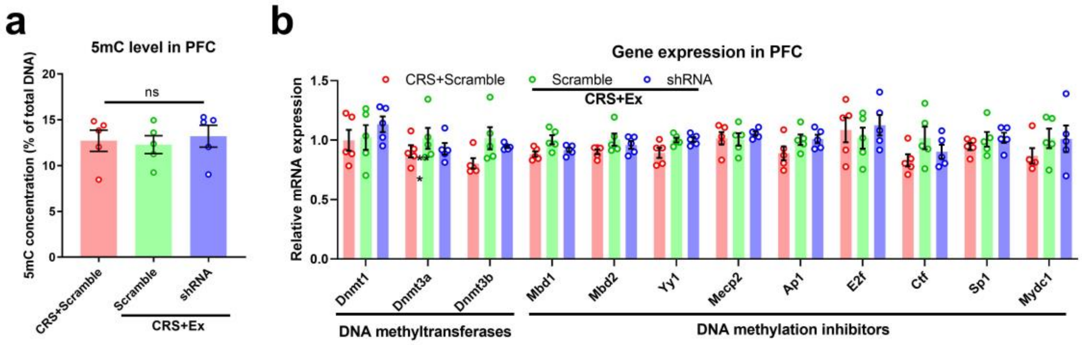

Extended Data Figure 6. Unchanged DNA methylation level. (a) No significant change of 5-methylcytosine (5mC) concentration in exercised mice or under hepatic *Mat1a* gene knockdown. One-way ANOVA, $F(2,12)=0.1690$, $P=0.847$. **(b)** The gene expression level of major DNA methyltransferases or methylation inhibitors remained unchanged, as suggested by multiple *t*-test. $N=5$ mice in each group in **(a-b)**. ns, no significant difference. All data are presented as mean± SEM.

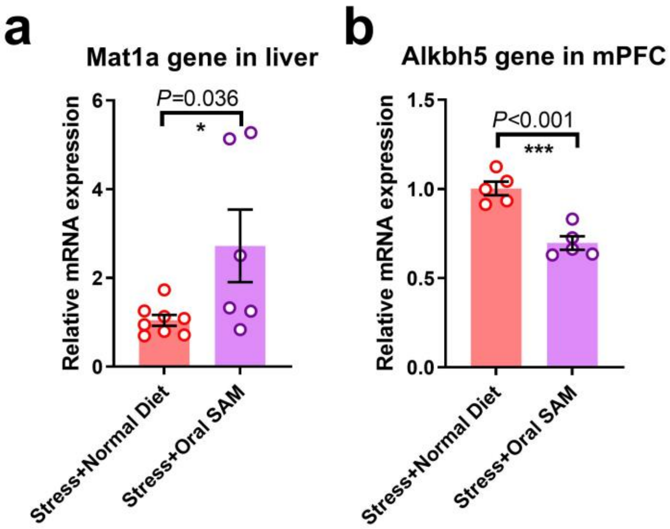

Extended Data Figure 7. Oral replenishment of methyl donor affects peripheral-central m6A homeostasis. (a) In CRS mice, 14-day oral SAM-enriched chows feeding remarkably increased liver expression of Mat1a. Two-sample *t*-test, $t(12)=2.355$, $P=0.036$. $N=8$ and 6 mice in normal diet and oral SAM groups, respectively. **(b)** Oral SAM-enriched chows feeding repressed central expression of RNA demethylase gene, Alkbh5. Two-sample *t*-test, $t(8)=5.687$, $P<0.001$. $N=5$ mice each group. All data are presented as mean±SEM.

Extended Data Table 2. Demographic information of MDD and healthy control people cohort.

**Healthy control group**			**MDD patient group**		
**Doc. No.**	**Gender**	**Age (yrs)**	**Doc. No.**	**Gender**	**Age (yrs)**
C201	Female	24	P203	Female	22
C202	Female	23	P208	Female	30
C203	Female	26	P214	Female	31
C204	Female	23	P215	Female	28
C206	Female	28	P228	Female	24
C101	Male	23	P238	Female	23
C102	Male	29	P106	Male	32
C176	Male	30	P113	Male	24
C196	Male	27	P128	Male	25
Total Male=4 (44%), Female=5 (56%)			Total Male=3 (33%), Female=6 (67%)		
Average age =25.9 yrs in Healthy, and 26.6 yrs in MDD, *P*=0.673 using 2-sample *t*-test.					

Extended Data Table 3. Sequence of primers in real-time qPCR.

**Target gene**	**Forward primer (5'-3')**	**Reverse primer (5'-3')**
Mat1a	GTGCTGGATGCTCACCTCAAG	CCACCCGCTGGTAATCAACC
Mat2a	GCTTCCACGAGGCGTTCAT	AGCATCACTGATTTGGTCACAA
Alkbh5	CGCGGTCATCAACGACTACC	ATGGGCTTGAACTGGAACTTG
Ythdf3	CATAGGGCAACAGAGGAAACAG	ATCTCCAGCCGTGGACCAT
Mettl3	CTGGGCACTTGGATTTAAGGAA	TGAGAGGTGGTGTAGCAACTT
Mettl14	CTGAGAGTGCGGATAGCATTG	GAGCAGATGTATCATAGGAAGCC
Wtap	GAACCTCTTCCTAAAAAGGTCCG	TTAACTCATCCCGTGCCATAAC
Fto	TCCTCAGAAGATGCCCTACTTG	CCCAACATTACCCAGCATGAAA
Ythdc1	GTCCACATTGCCTGTAAATGAGA	GGAAGCACCCAGTGTATAGGA
Ythdc2	ACCGACTAAGTCAATCTCTTGGT	AGGCTCCTAACAGCATGTTTTG
Ythdf2	GAGCAGAGACCAAAAGGTCAAG	CTGTGGGCTCAAGTAAGGTTC
Ythdf1	ACAGTTACCCCTCGATGAGTG	GGTAGTGAGATACGGGATGGGA
Gapdh	AGGTCGGTGTGAACGGATTTG	TGTAGACCATGTAGTTGAGGTCA

Extended Data Table 4. List of key reagents and sources.

Reagent or Resource name	Provider	Cat# or identifier#
**Antibody**		
GAPDH Antibody	CST	5174s
ALKBH5 Antibody	Abcam	ab195377
YTHDF3 Antibody	Abcam	ab220161
Rabbit Anti-c-Fos	Cell Signaling Technology	AB_2247211
TUBLIN Antibody	CST	2146s
MAT1A Antibody	Proteintech	12395-1-ap
m6A Antibody	Synaptic System	202-003
Streptavidin-Alexa Fluor 488	Thermo Fisher Scientific	AB_2315383
Rabbit Anti-CaMKIIa	Abcam	AB_305050
Alexa Fluor 488-conjugated Goat Anti-Rabbit	Jackson ImmunoResearch	AB_2338046
DyLight 594 Goat Anti-Mouse	Thermo Fisher Scientific	AB_1185569
DyLight 647 Goat Anti-Rabbit	Vector Laboratories	AB_2336420
**Bacterial and Virus Strains**		
rAAV2/9-CMV-rTTA-WPRE-hGH	Brain VTA, Wuhan, China	N/A
rAAV2/9 -EF1a-Alkbh5-P2A-mCherry-WPRE-hGH	Brain VTA, Wuhan, China	N/A
rAAV2/9 -EF1a- P2A-mCherry-WPRE-hGH	Brain VTA, Wuhan, China	N/A
rAAV2/9 -TRE3g-5'-miR30a-shRNA (Alkbh5)-3'-miR30a-CMV-mCherry-SV40	Brain VTA, Wuhan, China	N/A
rAAV2/9 -TRE3g-5'-miR30a-MCS-3'-miR30a-CMV-mCherry-SV40	Brain VTA, Wuhan, China	N/A
rAAV2/9 -TRE3g-5'-miR30a-shRNA (Ythdf3)-3'-miR30a-CMV-mCherry-SV40	Brain VTA, Wuhan, China	N/A
rAAV2/9 -EF1a-DIO-Alkbh5-P2A-mCherry-WPRES-hGH	Brain VTA, Wuhan, China	N/A
rAAV2/9 -Retro-DIO- mCherry-WPRES-hGH	Brain VTA, Wuhan, China	N/A
rAAV2/9 -CAG-FLEX-GGaMP6s- WPRE-SV40	Brain VTA, Wuhan, China	N/A
rAAV2/9-CMV-Cre-WPRE-hGH	Brain VTA, Wuhan, China	N/A
rAAV2/9-U6- shRNA(scramble) -CMV-mCherry-pA	Brain VTA, Wuhan,	N/A

	China	
rAAV2/8-TBG-mCherry-5'miR-30a-shRNA(Mat1a)-3'-miR30a-WPREs	Brain VTA, Wuhan, China	N/A
rAAV2/8-TBG-mCherry-5'miR-30a- 3'-miR30a-WPREs	Brain VTA, Wuhan, China	N/A
rAAV2/9-CaMKIIa-Alkbh5	Brain VTA, Wuhan, China	N/A
rAAV2/9-CaMKIIa-GCaMP6s	Taitool BioScience Co, Shanghai	N/A
rAAV2/9- CaMKIIa-hM3D(Gq)-mCherry	Taitool BioScience Co, Shanghai	N/A
rAAV2/9- CaMKIIa-hM4D(Gi)-mCherry	Taitool BioScience Co, Shanghai	N/A
rAAV2/9- CaMKIIa-mCherry	Taitool BioScience Co, Shanghai	N/A
**Chemicals, Peptides, and Recombinant Proteins**		
Clozapine N-oxide (CNO)	Sigma-Aldrich	Cat# C0832
Adenosylmethionine	Selleck	S5109
DOX	MCE	hy-n0565B
**Critical Commercial Assay Kits**		
PrimeScript^{TM} RT Regeat Kit	Takara Bioscience	Cat# RR420A
SYBR Premix Ex Taq^{TM}	Takara Bioscience	Cat# RR037A
BCA Protein Assay Kit	Biyuntian	p0012
SAM ELISA KIT	Jianglai Biological Technology Co., Ltd.	JL50114
Epiquik m6A RNA methylation quantification	Epigentek	p-9005
EZ-10 TOAL RNA MINI-RPEPS	BBI	B618583
SAM ELISA KIT	Biovision	f4541
